# Sugar-Based Polyesters: From Glux-Diol Synthesis to Its Enzymatic Polymerization

**DOI:** 10.1021/acsomega.5c08325

**Published:** 2026-01-08

**Authors:** Federico Acciaretti, Andrea Pasquale, Giacomo Lombardo, Giacomo Damonte, Simona Petroni, Luca Leuzzi, Marco Mangiagalli, Stefania Brocca, Alessandro Pellis, Laura Cipolla

**Affiliations:** † Department of Biotechnology and Biosciences, 9305 University of Milano-Bicocca, Piazza della Scienza 2, Milano I-20126, Italy; ‡ Department of Chemistry and Industrial Chemistry, 9302 University of Genova, Via Dodecaneso 31, Genoa (GE) 16146, Italy

## Abstract

This work details the synthesis of 2,4:3,5-di-*O*-methylene-d-glucitol (glux-diol), a bicyclic acetal derivative of d-glucose obtained from d-glucono-1,5-lactone, and also explores its subsequent polymerization to produce biobased polyesters. Key steps for the synthesis of glux-diol are (i) protection with paraformaldehyde, (ii) Fischer esterification, and (iii) reduction with lithium aluminum hydride (LiAlH_4_). A new purification method was developed to effectively remove inorganic salt byproducts, which can hinder polymerization. The sugar-based monomer was then copolymerized with C4–C10 dimethyl esters using *Candida antarctica* lipase B as a biocatalyst in Cygnet 2.0, a green, high-boiling solvent. The biocatalytic polycondensation produced oligomers with number-average molecular weights (*M*_n_) between 900 and 2200 g mol^–1^. These materials exhibited thermal stabilities ranging from 391 to 419 °C, with the specific temperature depending on the molecular weight, degree of polymerization, and the carbon chain length of the chosen diester. Overall, this integrated approach, which combines efficient sugar functionalization with biocatalysis, offers a promising pathway for the synthesis of novel biobased polyesters.

## Graphical abstract

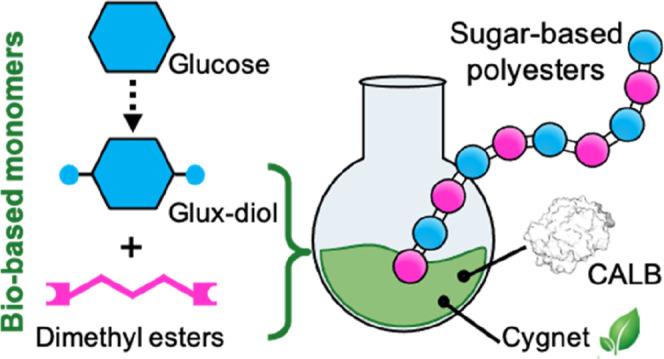

## 1 Introduction

Polyesters are a versatile class of polymers with a wide range of industrial applications, including textiles, furniture, regenerative medicine, drug delivery, packaging, automotive, and electronics. In recent years, both academic and industrial research have increasingly focused on biobased polyesters,^1^ aiming to replace fossil-derived monomers with renewable alternatives. This shift is driven not only by environmental concern but also by national and international policies promoting sustainability.^2^ Polyesters are typically synthesized from dicarboxylic acids (or their derivatives such as diesters and diacyl chlorides), diols, hydroxyacids or related compounds like hydroxyesters and lactones. Among the biobased building blocks under investigation, monosaccharides are particularly attractive due to their abundance from biomass and their rich stereochemistry, which offers opportunities to finely tune polymer properties.^3^ However, direct polymerization of unmodified monosaccharides is challenging because their polyhydroxylated skeleton hampers regioselective control during polymerization and often leads to products having a rather low thermal stability.^4^ To overcome these drawbacks, chemical modification, such as acetalization,^5^ is commonly employed to convert monosaccharides into more suitable monomers. In particular, *O*-protected alditol derivatives have been successfully incorporated into polyesters to improve their thermal properties, such as glass transition temperatures (*T*_g_).^6–9^ Among these, glucitol (sorbitol) has emerged as a valuable industrial biobased platform. For example, it can be used for the synthesis of 2,4:3,5-di-*O*-methylene-d-glucitol (glux-diol), which has been successfully copolymerized with common polyesters to produce copolymers of the glux-diol polyester with polyethylene terephthalate (PET), polybutylene terephthalate (PBT) and polybutylene sebacate, yielding materials with enhanced performance.

Beyond the origin of monomers, the sustainability of the polymerization process itself is a critical aspect of green polymer chemistry. Polyesters are typically synthesized from diols and dicarboxylic acids (or their esters) via polycondensation, using metal-based catalysts.^10^ However, these catalysts are generally unsuitable for polymerizing functional monomers like sorbitol and itaconic acid.^11,12^ In this context, biocatalysis offers a valuable alternative, allowing for milder reaction conditions,^13^ and compatibility with renewable, high-boiling organic solvents.^14^ Nonetheless, challenges remain in optimizing these enzymatic processes for industrial applications.

In this study, we revised and optimized a procedure for synthesizing glux-diol, a glucose derivative subsequently polymerized with C4–C10 dimethyl esters using *Candida antarctica* lipase B (CALB) in Cygnet 2.0, a cellulose-derived high-boiling solvent. This entirely biobased and sustainable approach provides access to novel polyesters under environmentally friendly conditions.

## 2 Experimental Section

### 2.1 Analytical Methods

#### 2.1.1 Thin Layer Chromatography (TLC)

Reactions were monitored by TLC on silica gel 60 F_254_ coated glass plates (Merck, Darmstadt, Germany) and visualized with a 45:45:10 H_2_O/EtOH/H_2_SO_4_ solution by heating at 100 °C until spots appear.

#### 2.1.2 Melting Point

Melting ranges were measured in duplicate with a Maplelab Scientific MPD-03 instrument (MPD-1-003, 230 V, 50 Hz, 80 W) on dried solids.

#### 2.1.3 Nuclear Magnetic Resonance (NMR) Spectroscopy

NMR spectra of intermediates and monomers were recorded at 400 MHz (^1^H) and 100.6 MHz (^13^C) with a Bruker Advance Neo instrument (Bruker, Billerica, MA, USA). Chemical shifts are reported in ppm referenced to residual solvent signal as an internal standard; *J* values are given in Hertz. For all the compounds, full assignments were based on 2D NMR techniques (^1^H–^13^C HSQC spectra).

NMR spectra of polymers were acquired at room temperature using a JEOL 400 MHz spectrometer, employing deuterated solvent CDCl_3_ with tetramethylsilane (TMS, 0.03%) as a reference. The ^1^H spectra were acquired at 400 MHz and the ^13^C spectra at 75 MHz. Alternatively, a Bruker AVANCE II 400 spectrometer was used with standard Bruker pulse programs. The samples were prepared by dissolving ∼10 mg of the polymer in 0.6 mL CDCl_3_. The spectra were reported with chemical shift (δ) in ppm normalized to the signal of TMS (0.00 ppm) on the *x*-axis and the signal intensity on the *y*-axis.

#### 2.1.4 Gel Permeation Chromatography (GPC)

Polymers were dissolved in THF at a concentration of ∼2 mg/mL and filtered through cotton wool packed into a 150 mm glass Pasteur pipet. The analysis was performed at 30 °C on an Agilent Technologies 1260 Infinity HPLC System equipped with a 17.369 6.0 mm ID × 40 mm LHHR-H, 5 μm guard column and an 18,055 7.8 mm ID × 300 mm L GMHHR-N, 5 μm TSK gel liquid Tosoh Bioscience chromatography column. CHCl_3_ served as the eluent, flowing at a rate of 1 mL/min for 20 min. An Agilent Technologies G1362A refractive index was used as the detector. The calibration curve was obtained using polystyrene standards in the 250–70,000 Da molecular weight range.

#### 2.1.5 Differential Scanning Calorimetry (DSC)

DSC analysis of the sugar-based polyesters was conducted using a Mettler Toledo DSC1 STARe System. A polymer sample of ∼5 mg was heated from 25 to 150 °C, held at 150 °C isothermally for 2 min, then cooled to −100 °C and heated again to 150 °C, always using a heating/cooling rate of 10 °C/min. Measurements were performed in an inert atmosphere under a constant N_2_ flow of 20 mL/min.

#### 2.1.6 Thermogravimetric Analysis (TGA)

TGA was performed using a TGA/DSC 1 Mettler Toledo “TGA/DSC1 STARe System” by placing polymer samples (10 mg) in 70 μL alumina crucibles. Measurements were carried out in the range from 25 to 800 °C by using a heating rate of 10 °C/min under a nitrogen flow of 80 mL/min.

### 2.2 Synthetic Procedures

#### 2.2.1 2,4:3,5-Di-*O*-methylene-d-gluconic Acid (**2**)

2.50 g of paraformaldehyde (83.26 mmol, 1.5 equiv)^15^ was added to 20 mL of 37% aq HCl (w/w) under magnetic stirring and the solution was heated to 60 °C until complete dissolution. Subsequently, 5.00 g of d-glucono-1,5-lactone (28.00 mmol, 1 equiv) was added to the hot paraformaldehyde solution and reacted until the starting material was completely consumed (as assessed by TLC), and a white solid precipitate was observed (typically within 5–6 h). The suspension was then cooled to room temperature (RT) and the solid was allowed to precipitate overnight. The reaction mixture was cooled for 2 h in an ice bath, after which the solid was collected by vacuum filtration using a Gooch funnel. Following overnight desiccation under vacuum compound **2** (4.25 g, 19.27 mmol) was obtained as a white solid, *R*_f_ = 0.2 (5:3:2 BuOH/EtOH/H_2_O). Mother liquors were brought to dryness, then additional product was recrystallized with hot Milli-Q H_2_O (0.53 g, 2.40 mmol), affording **2** in 77% overall yield.

#### 2.2.2 Methyl 2,4:3,5-Di-*O*-methylene-d-gluconate (**3**)

Acid **2** (3.00 g, 13.6 mmol) was added to 60 mL of a 0.3% (v/v) solution of conc. H_2_SO_4_ in dry MeOH; the heterogeneous mixture was refluxed under magnetic stirring until complete dissolution (approximately 2 h). When the starting material was almost completely consumed and no changes were observed by TLC (approximately 5 h), the mixture was cooled to room temperature and then neutralized by adding amount of Na_2_CO_3_ (0.34 g, 3.21 mmol) equivalent to the amount of H_2_SO_4_; salts were removed by vacuum filtration with a Büchner funnel. Methyl ester **3** was precipitated from the solution and filtered under vacuum using a Gooch funnel (white solid, 1.86 g, 7.91 mmol, *R*_f_ = 0.6, eluent 5:3:2 BuOH/EtOH/H_2_O). The mother liquors were evaporated to dryness, the solids were recrystallized from MeOH, and additional pure compound **3** (0.25 g, 1.06 mmol) was obtained. The total yield was 66%.

#### 2.2.3 1,6-Diacetyl-2,4:3,5-di-*O*-methylene-d-glucitol (**4**)

Compound **3** (0.50 g, 2.14 mmol, 1 equiv) was suspended in 15 mL of dry THF and the solution was refluxed until complete dissolution. LiAlH_4_ 1 M in dry THF (4.40 mL, 4.40 mmol, 4 equiv) was added slowly dropwise to the hot solution, and a precipitate immediately appeared. The reaction was stirred under reflux until complete conversion of the starting material (TLC 8:2 EtOAc/EtOH, *R*_f_ (reagent) = 0.6, *R*_f_ (product) = 0.4, about 4 h). The mixture was then cooled in an ice-bath and 1 mL of EtOAc was added dropwise to react any excess LiAlH_4_. Dry pyridine (3.50 mL, 43.45 mmol, 20 equiv) was added to the mixture at RT, followed by the dropwise addition of acetic anhydride (2.70 mL, 28.56 mmol, 7 equiv), and the addition of a catalytic amount of DMAP. The mixture was allowed to react with continuous magnetic stirring until the complete conversion of the starting material into the diacetylated products **4** (TLC 9:1 EtOAc/EtOH, *R*_f_ (reagent) = 0.2, *R*_f_ (product) = 0.8, about 17 h) was achieved. The emulsion formed by the aluminum and lithium salts was quenched by adding 30 mL of 5% aq HCl (v/v) and stirring with a glass rod until the mixture is clear. A liquid–liquid extraction was performed using 30 mL of EtOAc and then repeated two additional times. The combined organic phases were then dried over Na_2_SO_4_ and the salts were removed by gravity filtration. Mother liquors were evaporated to dryness, obtaining the crude product as a dense yellowish liquid. To remove the excess acetic acid formed as a byproduct during the acetylation reaction, 20 mL of DCM were added to the crude product, and then an acid–base extraction with 20 mL of saturated NaHCO_3_ was performed two times. Product **4** remains in the organic phase, which was dried over Na_2_SO_4_, filtered, and evaporated to dryness to obtain a brown solid (0.44 g). An analytical sample of compound **4** was recrystallized from hot EtOH.

#### 2.2.4 2,4:3,5-Di-*O*-methylene-d-glucitol (**5**)

Crude product **4** (0.44 g) was suspended in 7 mL dry MeOH at room temperature and stirred until complete dissolution. A catalytic amount of metallic sodium was added to the mixture and the reaction was carried out until the reagents were completely consumed (TLC, about 2 h). The ion exchanger Amberlite IR-120 resin was added to the crude product and the mixture was mildly stirred until the pH was slightly acidic (approximately half an hour). The consumed resin was then separated by gravity filtration. Pure glux-diol **5** precipitated from the solution as a white solid; filtration under vacuum using a Gooch funnel and desiccation under reduced pressure afforded 0.15 mg, (0.73 mmol) as a white solid, corresponding to an overall yield of 34% over three steps (reduction, acetylation, and deacetylation). An analytical sample of compound **5** was recrystallized from 95% aq EtOH (v/v).

### 2.3 Biocatalyzed Polyesters Synthesis

In a 25 mL round-bottom flask, 0.8 mmol of glux-diol and 0.8 mmol of aliphatic diester (C4, C6, C8 or C10) were added together with immobilized CALB (10% by weight of monomers) and Cygnet 2.0 (1.0 g) were heated to 85 °C at 400 rpm (12 mm stir bar) for 6 h. The system was then placed under a dynamic vacuum (20 mbar) for a further 88 h (total reaction time 96 h). For the workup procedure, 2 mL of THF was added to the reaction medium to solubilize the formed polymer, and the immobilized enzyme was removed by filtration through cotton packed into a 150 mm glass Pasteur pipet. The enzyme was further washed with 3 × 1 mL THF to remove all polymer. Subsequently, the polymer solution was added to 35 mL ice-cold water to induce precipitation. Samples were then filtered through a paper filter to discard the aqueous phase from the precipitated polymer. This procedure was repeated three times. Polymers were dried under vacuum before being fully characterized.

## 3 Results and Discussion

### 3.1 Synthesis of 2,4:3,5-Di-*O*-methylene-d-glucitol (Glux-Diol, **5**)

Two main synthetic strategies have been reported for the preparation of glux-diol, starting from biomass-derived monomers such as d-glucitol (sorbitol, Scheme S1, Supporting Information)^16^ or d-glucono-1,5-lactone (Scheme S2, Supporting Information).^17,18^ Among these, d-glucono-1,5-lactone appears more promising as it does not require an initial protection step to improve reaction regioselectivity. The synthetic strategy from d-glucono-1,5-lactone reported in the literature typically consists in a one-pot lactone hydrolysis and acetalization under acidic conditions to afford 2,4:3,5-di-*O*-methylene-d-gluconic acid (**2**), followed by Fischer esterification with methanol to yield methyl 2,4:3,5-di-*O*-methylene-d-gluconate (**3**), and subsequent ester reduction to the target glux-diol (**5**). However, reported procedures vary in the choice of acetalization reagent as well as in the nature and loading of the acid catalysts used in the first two steps (Tables S1 and S2, Supporting Information).^19–21^ Reproducing these literature procedures proved challenging; missing experimental details resulted in low yields and considerable product degradation. Additionally, the previously described workup to purify glux-diol from inorganic salts is limited by its low solubility in both aqueous and organic media. To address these issues, we revised the synthetic route to glux-diol from d-glucono-1,5-lactone (Scheme 1) and designed a novel workup procedure to remove inorganic byproducts and facilitate the isolation of pure glux-diol.

**Scheme 1:**
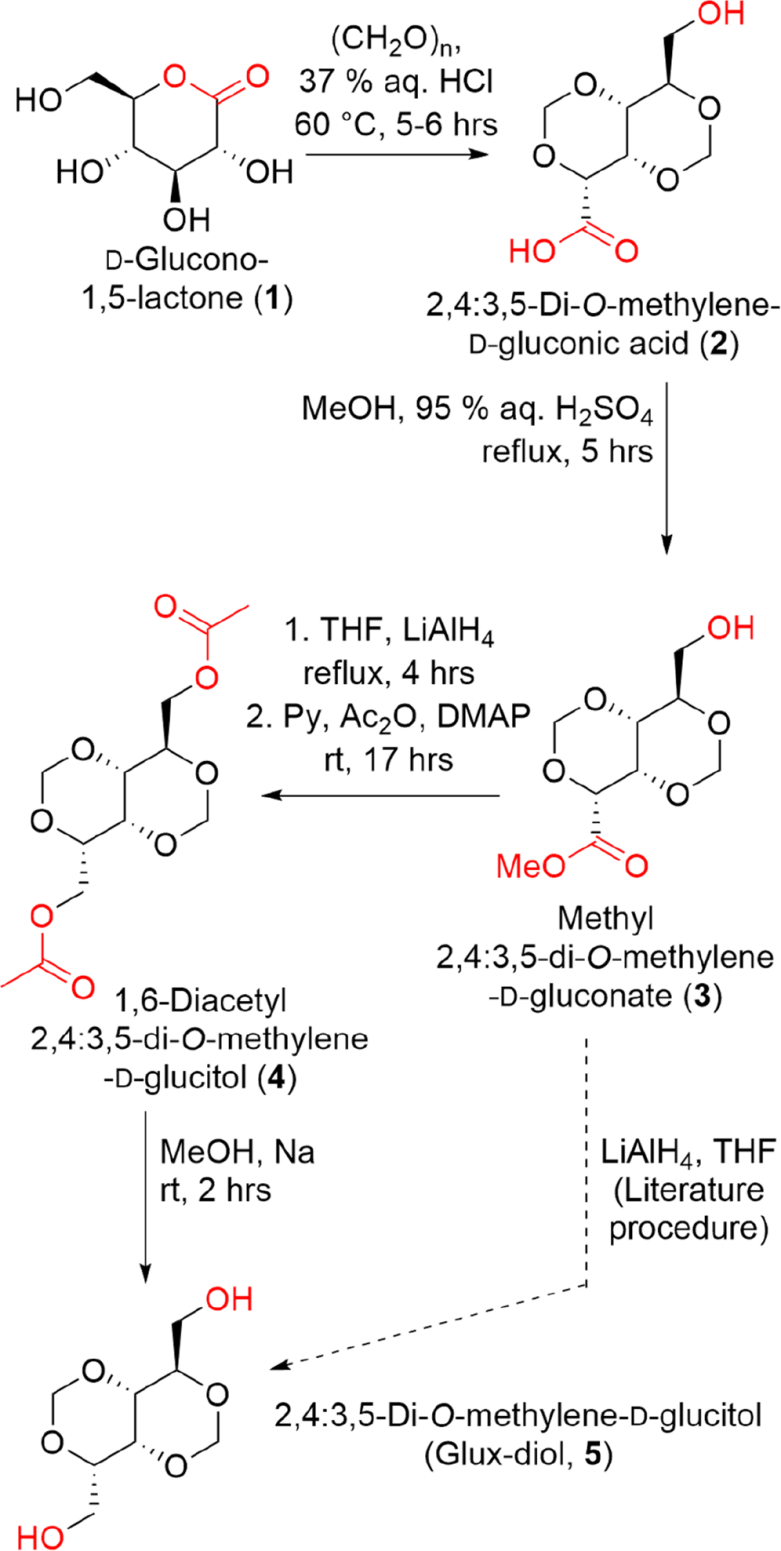
Synthetic Strategies from d-Glucono-1,5-lactone (**1**) to Glux-Diol (**5**)

Thus, the first step of the synthesis involves the acid-catalyzed acetalization of d-glucono-1,5-lactone with paraformaldehyde, accompanied by ring opening, to yield the d-gluconic acid derivative **2** (Scheme 1). Various reaction conditions—such as temperature, acid catalyst, reagent concentration—have been evaluated (Table S1, Supporting Information). Notably, the reagent concentration and the reaction temperature proved to be the most critical parameters. A temperature of 60 °C was found to be optimal, balancing the solubility of reagents with the thermal stability of the reaction components: lower temperatures hindered reagents dissolution, while higher temperatures led to product decomposition. Regarding concentration, the solvent volume needs to be sufficient to dissolve both d-glucono-1,5-lactone and paraformaldehyde, while simultaneously allowing compound **2** to reach saturation at the operating temperature and precipitate from the reaction medium. Solubility studies led to the following procedure (Table S1, Supporting Information): paraformaldehyde (1.5 equiv, 2.50 g) is first dissolved in aqueous HCl 37% w/w (8 mL/g paraformaldehyde) at 60 °C, followed by the addition of solid d-glucono-1,5-lactone (5.0 g, 1 equiv). Complete conversion into gluconic acid (**2**) is achieved within 5–6 h, as detected by TLC.

Conversion of the carboxylic group of compound **2** to its hydroxyl counterpart was achieved through Fischer esterification to the methyl ester intermediate **3**. Esterification is carried out in dry MeOH under reflux in the presence of 0.3% (v/v) H_2_SO_4_ in methanol. Key issue for the effectiveness of the reaction is the concentration of reagents in the reaction medium (Table S2, Supporting Information).

The reduction of methyl ester **3** with LiAlH_4_ in dry THF afforded glux-diol **5** along with inorganic byproducts derived from the hydride reagent. Efficient reduction requires an excess of reducing agent and minimal volume of solvent for dissolution of the starting material (**3**). Although LiAlH_4_ is widely used for large-scale reductions, the recovery of the desired alcohol is often complicated by the formation of large amounts of lithium and aluminum salts. While Fieser’s workup procedure is commonly used to remove these salts (Scheme 1, dashed arrow),^22^ it proved to be inadequate, mainly because of the poor water solubility of glux-diol, which caused its coprecipitation with inorganic salts. In addition, glux-diol also shows limited solubility in organic solvents. Collectively, these factors make purification of glux-diol a challenging task.

To improve extraction efficiency, methyl ester **3** was converted into diacetate **4** via a one-pot, two-step reduction-acetylation protocol (Scheme 1). Diacetate **4** exhibits an enhanced partition coefficient compared to glux-diol. Inorganic salts were removed by liquid–liquid extraction using 5% aqueous HCl and ethyl acetate, followed by the removal of residual acetic acid using saturated aq NaHCO_3_ and dichloromethane. The dried organic phase was concentrated, and the crude diacetate (**4**) underwent methanolysis in dry methanol with catalytic sodium (Supporting Information, Scheme S3). Neutralization of the reaction mixture with Amberlite IR-120 cation exchanger resin, followed by filtration and concentration to dryness, yielded crude glux-diol (**5**) devoid of inorganic salts. The final precipitation step in 95% (v/v) aqueous EtOH afforded analytically pure glux-diol without detectable traces of starting material.

The revised procedure proposed in this work yields glux-diol in high purity (>99% as determined by NMR), with an overall yield of 17% from d-glucono-1,5-lactone. This yield is lower than that reported by Marin and Muñoz-Guerra (34%), where the product was not submitted to any purification steps. As far as we know, our procedure is the first to include a clear and effective method for the complete removal of inorganic salts, a critical purification step that was not previously accounted for; thus, the proposed procedure enables the synthesis of glux-diol in an excellent purity grade, suitable for enzymatic polymerization.

### 3.2 Synthesis and Characterization of Glux-Diol-Derived Polyesters (**6**)

Although the chemical synthesis of glux-diol-based polyesters has already been reported using a classical approach based on mixing alkanedioic dialkyl esters with the glux-diol in a melt and using DBTO as the catalyst,^23^ a green and sustainable enzymatic approach for the synthesis of sugar-based polyesters starting from this monomer like the one presented in this manuscript has not yet been attempted. In this study, glux-diol was used in combination with a series of aliphatic dimethyl esters bearing linear alkyl chains of varying lengths (from C4 to C10) to enzymatically synthesize a family of polyesters. Immobilized lipase B from *Candida antarctica* was employed as the catalyst, and the reactions were carried out in the high-boiling, and green solvent Cygnet 2.0, a dioxolane-based compound derived from cellulose. Based on previous literature on the topic, this solvent is known to be suitable as replacement for the petrol-based and environmentally unfriendly solvent diphenyl ether for biocatalyzed polycondensation reactions of both conventional aliphatic and innovative cellulose-derived monomers.^24,25^ The polymerization was carried out using the protocol depicted in Scheme 2.

**Scheme 2:**
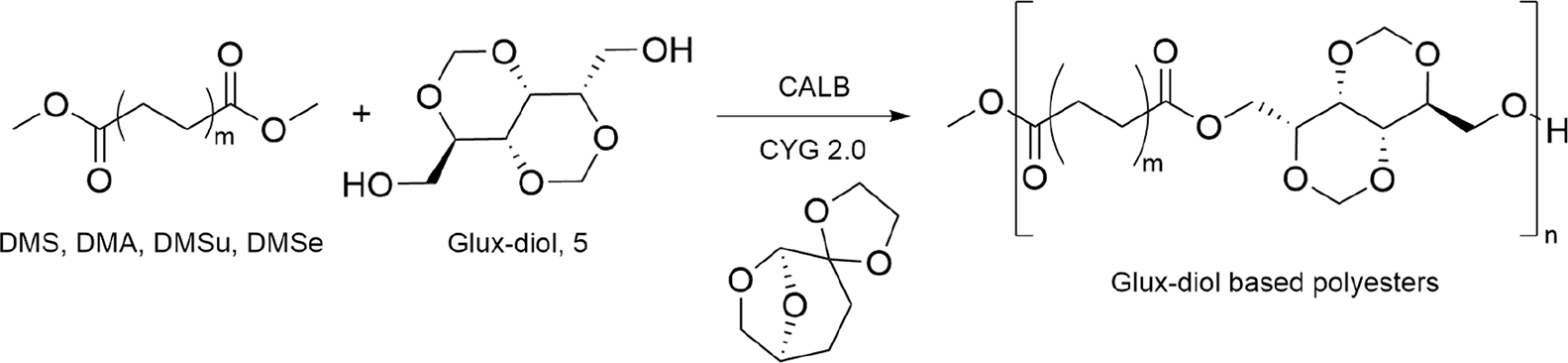
CALB-Mediated Polymerization of Glux-Diol with Different Aliphatic Dimethyl Esters: Dimethyl Succinate (DMS, *m* = 2), Dimethyl Adipate (DMA, *m* = 4), Dimethyl Suberate (DMSu, *m* = 6), Dimethyl Sebacate (DMSe, *m* = 8) in the High Boiling Cellulose-Derived Green Solvent Cygnet 2.0

A fully assigned ^1^H NMR spectra of a representative polymer is reported in Figure 1 (^1^H NMR characterization for all polymers is reported in the Supporting Information).

**Figure 1:**
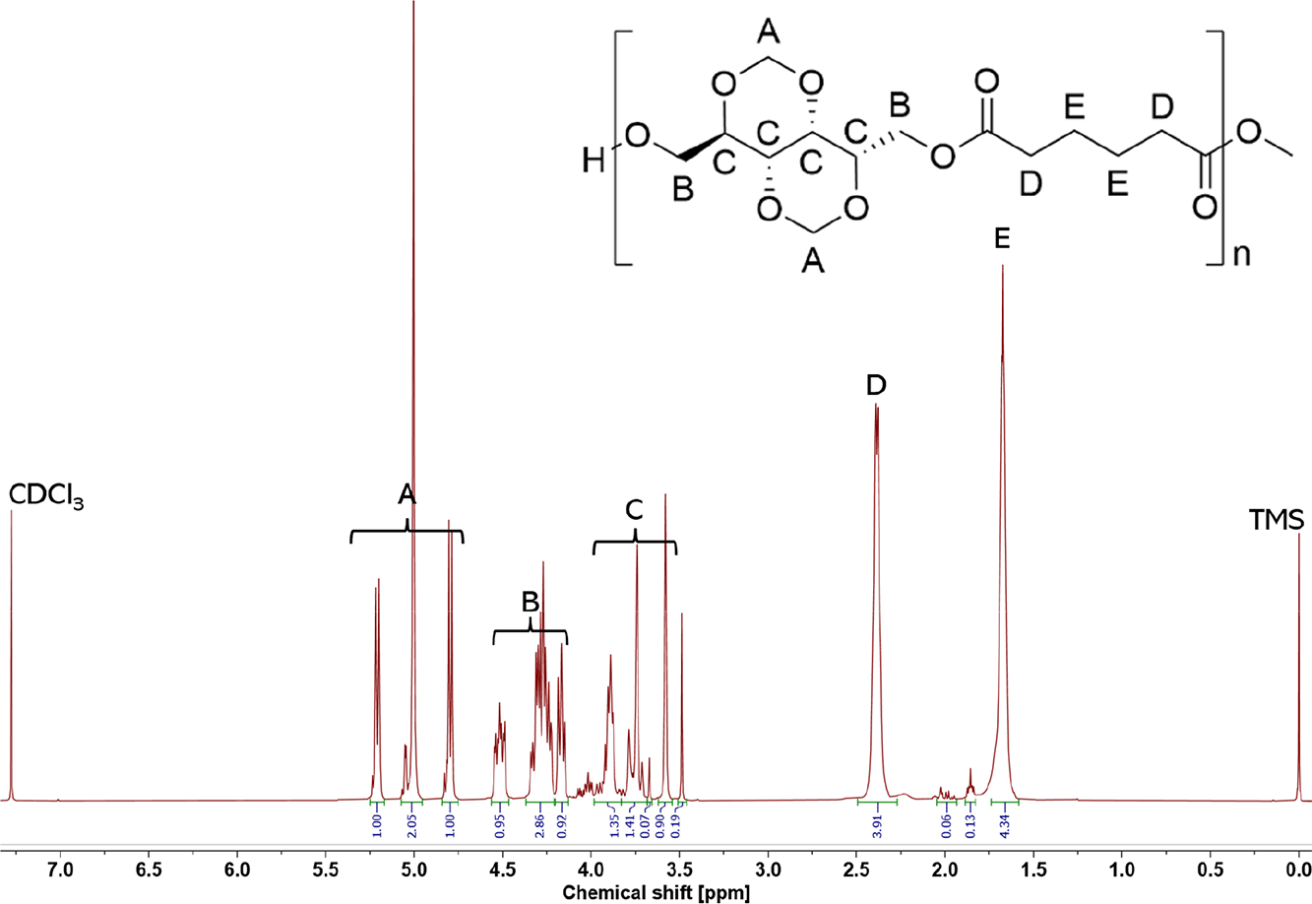
^1^H NMR: glux-diol and dimethyl adipate (DMA) based polyester.

The monomer conversion, calculated via ^1^H NMR considering the disappearance of the −CH_2_–CH_2_–OH groups of the diol and of the –OCH_3_ groups of the diesters, was excellent for all the tested diesters (>90%; Figure 2A), confirming the effectiveness of the enzymatic process. The polymerization yield, calculated gravimetrically, increased steadily with the length of the diester carbon chain, ranging from 40% for dimethyl succinate (C4, DMS) to 99% for dimethyl sebacate (C10, DMSe, Figure 2A). A similar trend was observed for the number-average molecular weight (*M*_n_) determined by GPC, which increased from 900 to 2200 g/mol as the chain length increased (Figure 2B). Thermal analysis of the resulting polymers revealed that their thermal stability improved with increasing diester chain length. Both the onset decomposition temperature (*T*_onset_) and the temperature corresponding to the maximum degradation rate (*T*_max_) increased progressively with the increasing diester chain length, ranging from 358 to 386 °C and from 391 to 419 °C, respectively (Figure 2 C,E). As expected, this trend was consistent with the simultaneous increase in the molecular weight and aliphatic content of the polymer chains. In fact, these two factors are known to act synergistically to enhance thermal stability by reducing both the number of terminal reactive groups and the overall ester bond density along the polymer backbone, which are otherwise prone to thermal degradation.^26,27^ In contrast, the glass transition temperature (*T*_g_) decreased with increasing chain length of the diester, dropping from 69 °C observed for the C4-derived polyester to 6 °C of the C10 analogue (Figure 2D,F). Additionally, the longer aliphatic spacer promoted the development of crystallization phenomena in both DMSu- and DMSe-derived polyesters (Figures 2D and S18). This effect, widely reported for aliphatic polyesters, is attributed to the enhanced ability of the aliphatic fraction to promote self-organization of the structure into crystalline domains. This behavior agrees with literature reports describing enzymatically synthesized aliphatic polyesters from rigid aromatic diols and flexible diacid components.^28^ These results demonstrate that glux-diol is a promising biobased monomer that can be efficiently exploited in enzymatic polycondensation reactions catalyzed by CALB to produce sugar-based polyesters.

**Figure 2:**
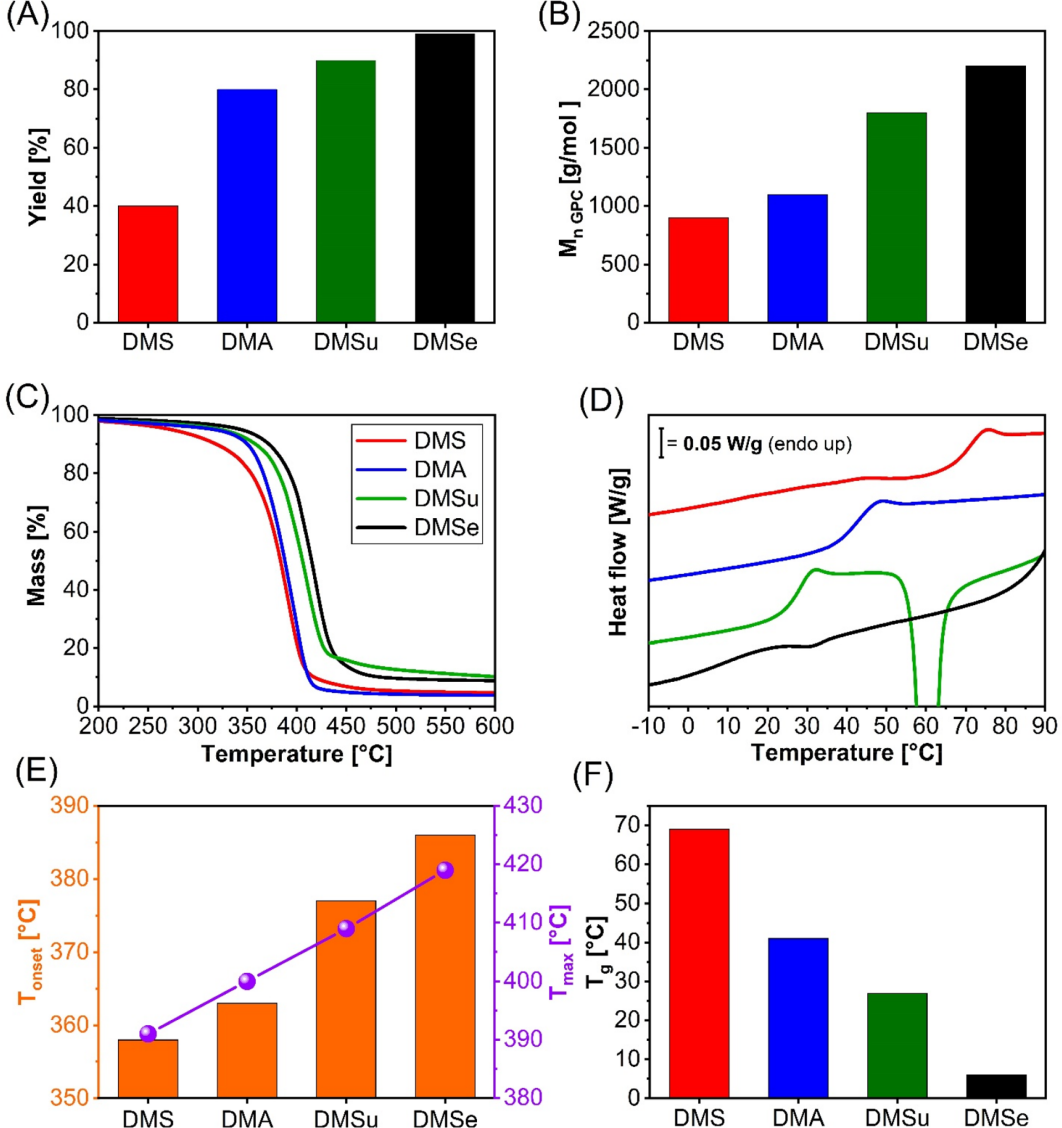
Enzymatic synthesis of glux-diol-based polyesters using Cygnet 2.0 as reaction solvent. Polymerization yield (A), number-average molecular weight M_*n*_ calculated via GPC (B), TGA thermograms (C), DSC curves (D), onset and maximum degradation rate temperatures (E) and *T*_g_ values (F).

## 4 Conclusion

Glux-diol was successfully synthesized with excellent purity, starting from d-glucono-1,5-lactone via a four-step procedure, achieving an overall yield of 17%. Our study led to consolidate and refine existing literature methods, resulting in a robust and reproducible synthesis protocol. The most critical challenge encountered was the purification of glux-diol from inorganic salts as byproducts of the reduction step, which significantly affected the final recovery step. Replacement of the classical Fieser’s workup with an acetylation/deacetylation strategy proved to be more effective for isolating pure glux-diol, a highly polar molecule, thus offering a valuable alternative for similar substrates. These methodological improvements, including the solvent systems and purification strategies described, are expected to facilitate the future scale-up and industrial translation of the process.

The enzymatic polycondensation of glux-diol with various aliphatic dimethyl esters, catalyzed by immobilized CALB in Cygnet 2.0, a cellulose-derived green solvent, yielded sugar-based polyesters with number-average molecular weights ranging from 900 to 2200 g/mol. The high monomer conversions, tunable physical properties, and use of a green solvent highlight the potential of this approach for the development of new sustainable materials with customizable performance profiles. Considering the natural abundance of glucose, overall, this work provides a comprehensive and scalable route for the valorization of glucose derivatives, such as glux-diol, in sustainable polymer chemistry.

## Supplementary Material



## Notes

Monomer synthesis—literature analysis and discussion; materials; product characterization; NMR spectra of intermediates, monomers, and polymers. All authors approved the final version of the manuscript before submission (PDF)

## §

Department of Chemistry, Material and Chemical Engineering “Giulio Natta”, Politecnico di Milano, Piazza Leonardo da Vinci 32, 20133, Milano, Italy

## ∥

Istituto Istruzione Superiore Statale “Luigi Castiglioni” Via Giuseppe Garibaldi, 115, 20812 Limbiate, Italy.
