# Supplementary material for: Sugar-Based Polyesters: From Glux-Diol Synthesis to Its Enzymatic Polymerization
Source: ACS Omega. 2026 Jan 8;11(2):2722–9. doi: 10.1021/acsomega.5c08325 (PMC12824748; doi:10.1021/acsomega.5c08325)
Supplement: Supplementary file 1 [file ao5c08325_si_001.pdf]

Supporting information

Sugar-based polyesters: from glux-diol synthesis to its enzymatic polymerization

Federico Acciaretti[a,§], Andrea Pasquale[b], Giacomo Lombardo[b], Giacomo Damonte[b], Simona Petroni[a,¶], Luca Leuzzi[a], Marco Mangiagalli[a], Stefania Brocca[a], Alessandro Pellis[b], and Laura Cipolla[a,*]

[a]Department of Biotechnology and Biosciences, University of Milano - Bicocca, Piazza della Scienza 2, I-20126 Milano, Italy.
[b]Department of Chemistry and Industrial Chemistry, University of Genova, Via Dodecaneso 31, 16146, Genoa (GE), Italy.

*Corresponding author: Prof. Laura Cipolla, Department of Biotechnology and Biosciences, University of Milano - Bicocca, Piazza della Scienza 2, I-20126 Milano, Italy. Email: laura.cipolla@unimib.it

§ Present address: Department of Chemistry, Material and Chemical Engineering “Giulio Natta”, Politecnico di Milano, Piazza Leonardo da Vinci 32, 20133, Milano, Italy.
¶ Present address: Istituto Istruzione Superiore Statale "Luigi Castiglioni" Via Giuseppe Garibaldi, 115, 20812 Limbiate, Italy

Table of contents

1. **Monomer synthesis - literature analysis and discussion** **p. 2**
2. **Materials** **p. 8**
3. **Product Characterization** **p. 8**
4. **NMR spectra of intermediates, monomers, and polymers** **p. 10**

1. Monomer synthesis - literature analysis and discussion

Glux-diol from D-glucitol

The synthesis of glux-diol from D-glucitol involves three synthetic steps: initial protection of the primary hydroxyl groups, acetalization of the secondary hydroxyl groups, and final deprotection (Scheme S1). Even though it comprises just three steps, the primary drawback is the low yield (11%, Scheme S1) of the initial step. This is attributed to the formation of regioisomers, stemming from the challenging control of regioselective acylation, particularly over primary versus secondary hydroxyl groups. Consequently, this strategy was not considered in the present work.

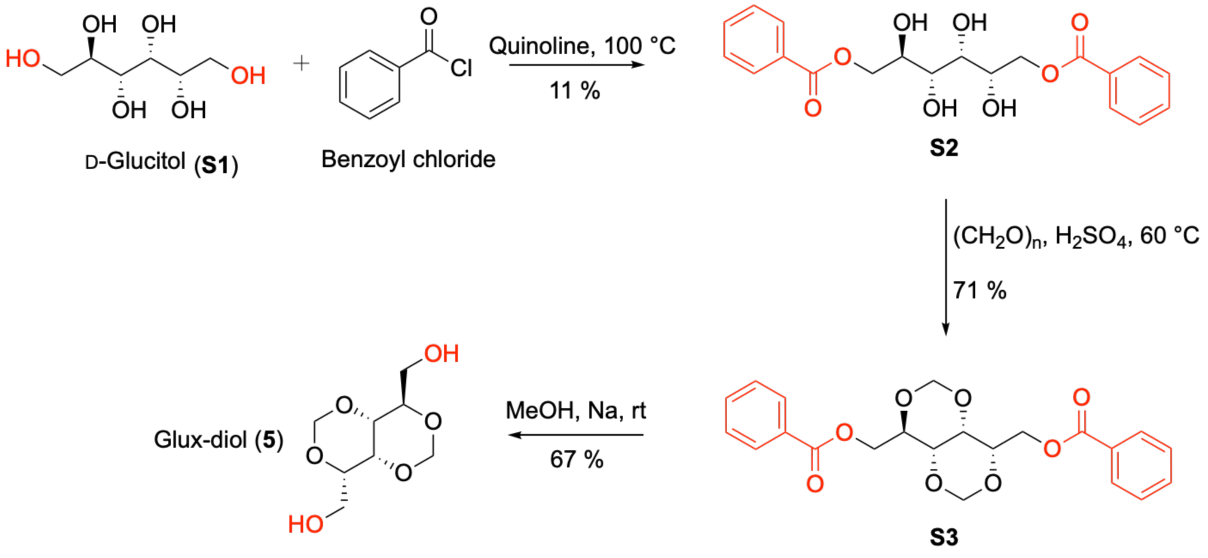

Scheme S1. Reported synthetic strategy for 2,4:3,5-di-*O*-methylene-D-glucitol from D-glucitol.[1]

Glux-diol from D-gluconolactone

An alternative strategy described in the literature uses D-glucono-1,5-lactone (Scheme S2). The absence of an initial protection step makes this route a potentially advantageous strategy, which we've considered and developed in this work. Every step of the synthesis for converting D-glucono-1,5-lactone into glux-diol is detailed in the following.

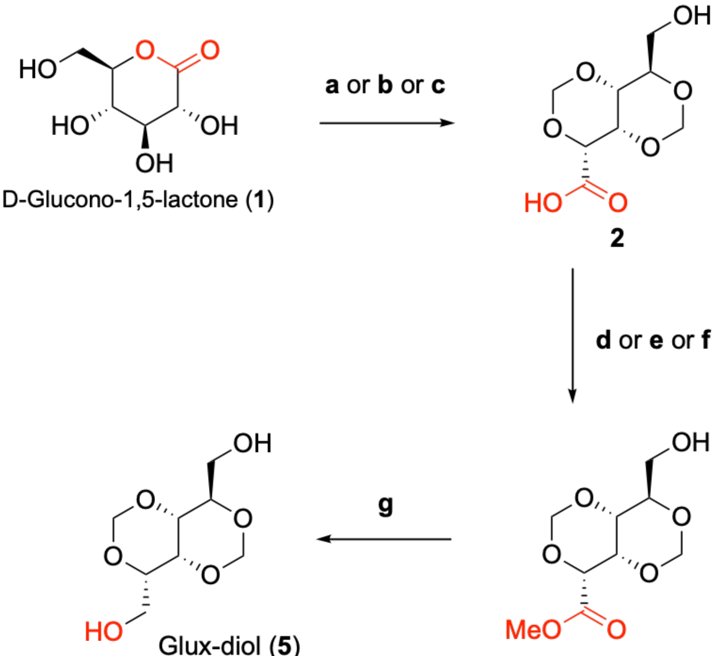

Scheme S2. General synthetic strategy towards 2,4:3,5-Di-*O*-methylene-D-glucitol (**5**) from D-glucono-1,5-lactone (**1**), as reported in the literature. a) Trioxane (37 % aq, HCl (w/w), 24 hrs, room temperature; work-up: dilution, filtration and recrystallization, 59 % yield.[2] b) Trioxane, 37 % aq. HCl (w/w), 24 h, room temperature; work-up: washing with toluene and filtration, 85 % yield.[3] c) Paraformaldehyde, 37 % aq. HCl (w/w), 1 hrs, reflux; work-up: filtration, 80 % yield.[4] d) MeOH, 95 % aq. H_2SO_4 (w/w), 23 hrs, reflux; work-up: filtration, concentration of the mother liquors and recrystallization, 87 % yield.[5] e) MeOH, *p*-toluenesulfonic acid, 12 hrs, reflux; work-up: filtration, concentration of the mother liquors and recrystallization, 90 % yield].[3] f) MeOH, 95 % aq. H_2SO_4 (w/w), reflux; work-up: filtration and concentration of the mother liquors, 70 % yield.[4] g) THF, $LiAlH_4$, 24 hrs, reflux; work-up: Fieser procedure, 60 % yield.[4,6,]

2,4:3,5-di-*O*-methylene-D-gluconic acid (2)

The initial step of the synthesis involves the acid-catalyzed acetalization of D-glucono-1,5-lactone either with trioxane (Entry 1 and 2, Table S1), or paraformaldehyde (Entry 3, Table S1). This reaction concurrently leads to the opening of the lactone's cyclic structure. Complete dissolution of all reagents is required for this stage. Literature procedures report that 0.5 mL of aqueous HCl (37 % *w/w*) allows the complete dissolution of 100 mg of D-glucono-1,5-lactone and the reported amount of paraformaldehyde (Table S1, Entry 3). In our hands, preliminary experiments showed that this volume was inadequate for dissolving reagents, at the reported temperatures. Thus, we initially focused on the study of individual solubilities of the two reagents at 60 °C. While 0.50 mL of solvent was sufficient to dissolve 100 mg of D-glucono-1,5-lactone, 1.00 mL was needed for 100 mg of paraformaldehyde. Therefore, paraformaldehyde dictated the necessary solvent volume. After several attempts,

the optimal conditions for dissolving both reagents involved first dissolving paraformaldehyde (1.5 equiv.) in 8 mL of 37 % aq. HCl per gram of paraformaldehyde at 60 °C, then adding portionwise solid D-glucono-1,5-lactone.

Monitoring the reaction with thin layer chromatography (TLC) allowed to evaluate the complete conversion of the reagents into products, the formation of by-products that remained in the mother liquor, and to qualitatively check the purity of the isolated solid. In previous papers, TLC was not considered a practical approach for reaction analysis.

Table S1. Summary of reported reaction conditions for the synthesis of 2,4:3,5-di-*O*-methylene-D-gluconic acid (**2**) from D-glucono-1,5-lactone (**1**).

Entry	T (°C)	Time (hrs)	Lactone (1)	Formaldehyde equivalents	37 % aq. HCl (% m/v)[b]	Yield (%)	Lit.
1	rt	72	17.80 g<br>0.10 mol<br>1 equiv.	Trioxane<br>9.00 g<br>0.10 mol<br>1.5 equiv.	25.00 mL<br>(36 %)	59	[2]
2	rt	24	4.50 g<br>25.30 mmol<br>1 equiv.	Trioxane<br>2.30 g<br>25.30 mmol<br>1.5 equiv.	20.00 mL<br>(11.5 %)	85	[3]
3	Reflux	1	30.00 g<br>0.17 mol<br>1 equiv.	Paraformaldehyde[a]<br>30.00 g<br>1.00 mol<br>3 equiv.	42.00 mL<br>(71.4 %)	80	[4]
4	60 °C	5-6	5.00<br>28.00 mmol<br>1 equiv.	Paraformaldehyde[a]<br>2.50 g<br>83.26 mmol<br>1.5 equiv.	20.00 mL<br>(12.5 %)	77	This study

[a]Molar amount of paraformaldehyde is expressed in terms formaldehyde monomers, calculated dividing mass of weighted paraformaldehyde by repeating unit mass (-CH_2O-, 30.026 g/mol); 1 equiv. of formaldehyde is considered as the amount needed to react with 1 mole of D-glucono-1,5-lactone to yield compound **2**; hence, 1 equiv. of formaldehyde equals 2 moles, since two moles of formaldehyde are needed to react with 1 mole of lactone to obtain compound **2**.
[b]For sake of clarity, m/v % concentration of the acetalization reagent in 37 % aq. HCl is reported.

Methyl 2,4:3,5-di-*O*-methylene-D-gluconate (3)

In the Fischer esterification, an equilibrium reaction, the concentration of the reaction mixture is a key parameter in maximizing the percent conversion. Literature reports that 8.5 mL of MeOH completely solubilizes gluconic acid derivative **2** (Table S2, Entry 3). We observed that in these conditions compound **2** is only partially soluble and a suspension is formed, making the reaction more difficult to take place. The optimal condition was determined to be 20.00 mL of dry methanol per gram of compound **2**, refluxed for approximately 3 h until a clear solution was achieved. The Fischer esterification was carried out for 5 h in the presence of 0.3% H_2SO_4 as catalyst in dry MeOH (v/v). The work-up procedure consists in a first step of neutralisation of the acidic solution with Na_2CO_3, followed by crystallization of the product from the mother liquors.

Table S2. Summary of reported reaction conditions for the synthesis of methyl 2,4:3,5-di-*O*-methylene-D-gluconate (**3**) from 2,4:3,5-di-O-methylene-D-gluconic acid (**2**).

Entry	T (°C)	Time (hrs	Gluconic acid (2)	MeOH (% m/v of **2**)	Catalyst quantity	Yield (%)	Lit.
1	Reflux	23	400.00 g (1810 mmol)	3.50 L (11.4 %)	95 % aq. H_2SO_4 (w/w) 4.00 mL	87	[5]
2	Reflux	12	3.00 g (13.6 mmol)	not reported	PTSA Catalytic amount	90	[3]
3	Reflux	n.d.	30.00 g (136 mmol)	250.00 mL (12 %)	95 % aq. H_2SO_4 (w/w) 0.75 mL	70	[4]
4	Reflux	5	3.00 g (13.6 mmol)	60.00 mL (5 %)	95 % aq. H_2SO_4 (w/w) 0.18 mL	66	This study

2,4:3,5-di-*O*-methylene-D-glucitol (5)

The conversion of methyl ester **3** to glux-diol via hydride reduction, typically with lithium aluminum hydride ($LiAlH_4$), often necessitates excess reagent. In line with literature reports,[4] a 1:2 molar ratio of methyl 2,4:3,5-di-O-methylene-D-gluconate (**3**) to $LiAlH_4$ was found to be effective and was successfully replicated in the present work. While effective, the use of reducing hydrides carries several drawbacks, notably the significant amphipathic colloidal salt formation that may hinder product isolation. While the Fieser workup[6] is commonly employed to recover glux-diol from the reaction medium, in our hands it failed to yield a pure compound suitable for subsequent polymerization. Alternative purification techniques were attempted to separate salts from glux-diol, including solid-liquid extraction with different solvents (MilliQ H_2O, EtOH), liquid-liquid extraction with hydrophobic organic solvents (CH_2Cl_2, $CHCl_3$, EtOAc), and the recrystallisation of diol **5** from different organic solvents. Unfortunately, these methods proved unsatisfactory, yielding only small quantities of pure glux-diol. To address these challenges, we propose a one-pot, two-step acetylation/deacetylation sequence, combined with appropriate liquid extraction steps. This process is schematically depicted in Scheme **S3**.

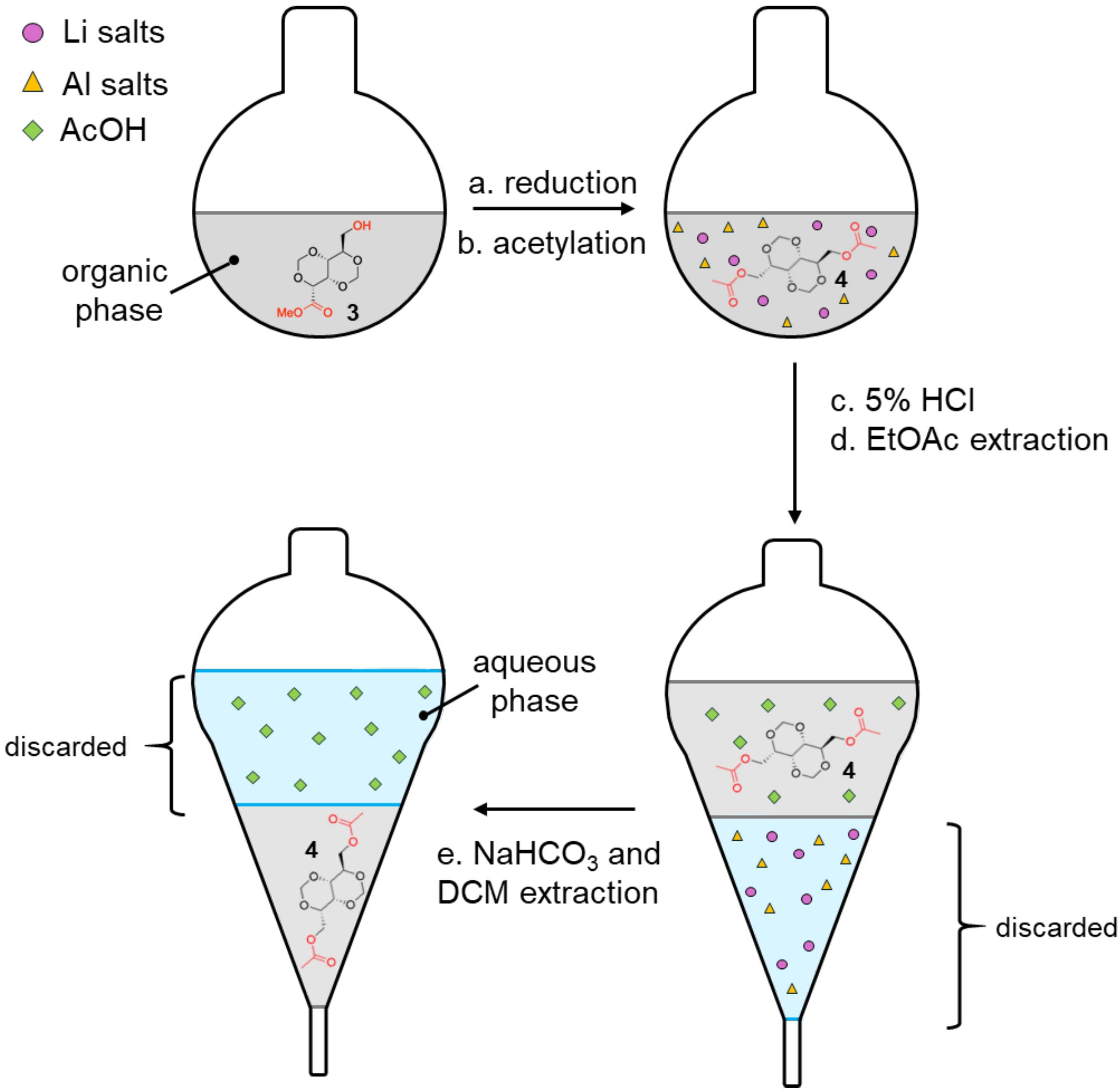

Scheme S3. Schematic diagram of the workflow of the two steps one-pot reduction-acetylation (**a-b**); **c.** liquid-liquid extraction of the reaction mixture with 5% aq. HCl (w/w) and EtOAc: lithium chloride, aluminium chloride and acetic acid were formed; **d.** Extraction of product **4** and subsequent concentration to dryness under vacuum (acetic acid residues are present in the organic phase); salts were retained by the aqueous phase and discarded. **e.** Acid-base extraction with DCM and saturated aq. $NaHCO_3$: sodium acetate was extracted in the aqueous phase and removed, while product **4** remained in the organic phase, which is dried over Na_2SO_4, filtered and concentrated to dryness.

2. Materials

D-Glucono-1,5-lactone (CAS 90-80-2, 99%), paraformaldehyde (CAS 30525-89-4, 89-93%), acetic anhydride (CAS 108-24-7, 99%), dry pyridine (CAS 110-86-1), 4-(dimethylamino)pyridine (CAS 1122-58-3, DMAP), 95% aq. H_2SO_4 (w/w), dry tetrahydrofuran (CAS 16853-85-3, THF), butanol (BuOH), Na_2SO_4 (CAS 7757-82-6), ion exchanger Amberlite IR-120 resin, deuterated dimethyl sulfoxide (DMSO-d6) and deuterated chloroform ($CDCl_3$) were purchased from Merck (Merck, Darmstadt, Germany). Lithium aluminium hydride ($LiAlH_4$) 1 M in dry THF, 37% aq. HCl (w/w), dry methanol (MeOH) and Na_2CO_3 (CAS 497-19-8) were purchased from Acros Organics (Fisher Scientific Italia). Metallic sodium (CAS 7440-23-5), sodium bicarbonate ($NaHCO_3$), ethyl acetate (EtOAc), and ethanol (EtOH) were purchased from Thermo Scientific Chemicals (Fisher Scientific Italia). Dichloromethane (DCM) was purchased from Honeywell Riedel-de-Haën. Dry pyridine was purchased from VWR International S.r.l. (Milano, Italia).
Dimethyl succinate (DMS) (CAS 106-65-0, 99%, Tokyo Chemical Industry), dimethyl adipate (DMA) (CAS 627-93-0, 99%, Tokyo Chemical Industry) , dimethyl suberate (DMSu) (CAS 1732-09-8, >98%, Tokyo Chemical Industry) dimethyl sebacate (DMSe) (CAS 106-79-6, >98%, Tokyo Chemical Industry), THF (CAS 109-99-9, 99%, VWR Chemicals), Lipase B from *Candida antarctica* immobilized on acrylic resin (also known as Novozyme 435) was purchased from Merck (code L4777). This preparation is a Novozym product where the enzyme is expressed in *Aspergillus niger* and the final preparation as a PLU activity of ≥5,000 U/g. Cygnet 2.0 was synthesized and characterized as previously reported by *Warne et al.* [7]

3. Product Characterization

2,4:3,5-Di-*O*-methylene-D-gluconic acid (2)
M.p. 217.3-219.5 °C (lit. 217.4-219.4 °C);[2] ^{1}H NMR (400 MHz, DMSO-d6): δ 12.86 (s, 1H, COO*H*), 5.04, 4.76 (ABq, 2H, J 6.3 Hz, O-CH_2-O), 4.94, 4.77 (ABq, 2H, J 6.2 Hz, O-CH_2-O), 4.99 - 4.85 (m, 1 H, O*H*), 4.40 (d, 1H, $J_{2,3}$ 2.0 Hz, H-2), 4.10 (br s, 1H, H-3), 3.78 (br s, 1H, H-4), 3.78 - 3.64 (m, 3H, H-5, H-6a, H-6b). ^{13}C NMR (101 MHz, DMSO-d6): δ 169.32 (s, C-1), 91.72 (q, O-*C*H_2-O), 88.03 (q, O-*C*H_2-O), 76.63 (d, C-5), 75.98 (d, C-2), 70.61 (d, C-4), 68.42 (d, C-3), 59.07 (q, C-6).

Methyl 2,4:3,5-di-*O*-methylene-D-gluconate (3)
M.p. 152.2-153.1 °C (lit. 151-152 °C);[5] ^{1}H NMR (400 MHz, $CDCl_3$): δ 5.26, 4.78 (ABq, 2H, J 6.5 Hz, O-CH_2-O), 5.03 (s, 2H, O-CH_2-O), 4.33 (d, 1H, $J_{2,3}$ 2.0 Hz, H-2), 4.14 (br s, 1H, H-3), 4.02 - 3.99 (m, 1H, H-5), 3.94 (dd, 1H, $J_{6a,6b}$ 11.4 Hz, $J_{5,6a}$ 6.4 Hz, H-6a), 3.86 (dd, 1H, $J_{6b,6a}$ 11.4 Hz, $J_{5,6b}$ 5.3 Hz, H-6b), 3.82 (br s, 4H, H-4, OCH_3). ^{13}C NMR (101 MHz, $CDCl_3$): δ 167.98 (s, C-1), 92.35 (q, O-*C*H_2-O), 88.65 (q, O-*C*H_2-O), 76.63 (d, C-2), 76.43 (d, C-5), 71.54 (d, C-4), 67.55 (d, C-3), 60.72 (q, C-6), 52.65 (q, O*C*H_3).

1,6-Diacetyl-2,4:3,5-di-*O*-methylene-D-glucitol (4)

M.p. 108-110.5 °C (lit. 114-115 °C);[8] ^{1}H NMR (400 MHz, $CDCl_3$): δ 5.21, 4.80 (ABq, 2H, J 6.5 Hz, O-C*H₂*-O), 5.01, 4.99 (ABq, 2H, J 6.3 Hz, O-C*H₂*-O), 4.46 (dd, 1H, $J_{6a,6b}$ 11.8 Hz, $J_{6a,5}$ 7,3 Hz, H-6a), 4.31 (dd, 1H, $J_{1a,1b}$ 11.8 Hz, $J_{1a,2}$ 4.7 Hz, H-1a), 4.28 - 4.22 (m, 2H, H-1b, H-6b), 4.17 - 4.14 (m, 1H, H-5), 3.89 - 3.85 (m, 1H, H-2), 3.72 (br s, 1H, H-3), 3.56 (br s, 1H, H-4), 2.10 (s, 3H, CH_3), 2.09 (s, 3H, CH_3). ^{13}C NMR (101 MHz, $CDCl_3$): δ 170.81 (s, *C*=O), 170.57 (s, *C*=O), 92.89 (q, O-CH_2-O), 88.37 (q, O-CH_2-O), 75.49 (d, C-2), 73.64 (d, C-5), 70.89 (d, C-4), 67.22 (d, C-3), 63.25 (q, C-1), 60.61 (q, C-6), 20.86 (q, CH_3), 20.80 (q, CH_3).

2,4:3,5-Di-*O*-methylene-D-glucitol (5)

R_f = 0.2 (9:1 EtOAc:EtOH); M.p. 193-196.3 °C (lit. 192-193 °C);[1] ^{1}H NMR (400 MHz, DMSO-d6): δ 5.00, 4.72 (ABq, 2H, J 6.2 Hz, O-CH_2-O), 4.90, 4.78 (ABq, 2H, J 6.3 Hz, O-CH_2-O), 4.91 - 4.88 (m, 1H, O*H*), 4.73 - 4.71 (m, 1H, O*H*), 3.79 (br s, 1H, H-3), 3.74 - 3.65 (m, 3H, H-5, H-6a, H-6b), 3.65 (br s, 1H, H-4), 3.63 - 3.60 (m, 1H, H-2), 3.50 (dd, 1H, $J_{1a,1b}$ 11.1 Hz, $J_{1a,2}$ 5.9 Hz, H-1a), 3.45 (dd, 1H, $J_{1b,1a}$ 11.1 Hz, $J_{1b,2}$ 6.7 Hz, H-1b). ^{13}C NMR (101 MHz, DMSO-d6): δ 92.40 (q, O-CH_2-O), 87.91 (q, O-CH_2-O), 78.62 (d, C-2), 76.63 (d, C-5), 70.90 (d, C-4), 67.21 (d, C-3), 60.04 (q, C-1), 58.81 (q, C-6).

Cygnet 2.0 (dioxolane cygnet)

^{1}H-NMR (400 MHz, $CDCl_3$): δ 7.26 ($CDCl_3$), 5.00 (s, 1H, H-A), 4.53 (m, 1H, H-B), 4.03 (m, 2H, H-Ca, H-Cb), 3.91 (m, 3H, H-Da, H-Db, H-Ea), 3.81 (m, 1H, H-Eb), 2.01 (m, 2H, H-Fa, H-Fb), 1.65 (m, 2H, H-Ga, H-Gb).

Glux-diol and dimethyl succinate (DMS) based polyester

^{1}H-NMR (400 MHz, $CDCl_3$): δ 7.26 ($CDCl_3$), 5.20, 4.99, 4.77 (ABq, 4H, H-A O-CH_2-O), 4.49 (m, 1H, Cygnet 2.0), 4.26, 4.15 (m, 4H, H-B), 3.86, 3.71, 3.65, 3.55 (m, 4H, H-C) 2.32 (m, 4H, H-D), 1.62 (H_2O).

Glux-diol and dimethyl adipate (DMA) based polyester

^{1}H-NMR (400 MHz, $CDCl_3$): δ 7.26 ($CDCl_3$), 5.21, 5.00, 4.80 (ABq, 4H, H-A O-CH_2-O), 4.52 (m, 1H, Cygnet 2.0), 4.27, 4.17 (m, 4H, H-B), 3.89, 3.76, 3.58, 3.49 (m, 4H, H-C) 2.39 (m, 4H, H-D), 1.67 (m, 4H, H-E).

Glux-diol and dimethyl suberate (DMSu) based polyester

^{1}H-NMR (400 MHz, $CDCl_3$): δ 7.26 ($CDCl_3$), 5.19, 5.01, 4.78 (ABq, 4H, H-A O-CH_2-O), 4.49 (m, 1H, Cygnet 2.0), 4.30, 4.15 (m, 4H, H-B), 3.88, 3.75, 3.68, 3.59 (m, 4H, H-C) 2.67 (m, 4H, H-D), 1.56 (H_2O), 1.24 (m, 4H, H-E).

Glux-diol and dimethyl sebacate (DMSe) based polyester

^{1}H-NMR (400 MHz, $CDCl_3$): δ 7.26 ($CDCl_3$), 5.20, 4.99, 4.77 (ABq, 4H, H-A O-CH_2-O), 4.47 (m, 1H, Cygnet 2.0), 4.31, 4.24, 4.15 (m, 4H, H-B), 3.86, 3.72, 3.65, 3.55 (m, 4H, H-C), 2.33 (m, 5H, H-D), 1.61 (H_2O), 1.29 (m, 11H, H-E, H-F, H-G).

4. NMR spectra of intermediates, monomers, and polymers

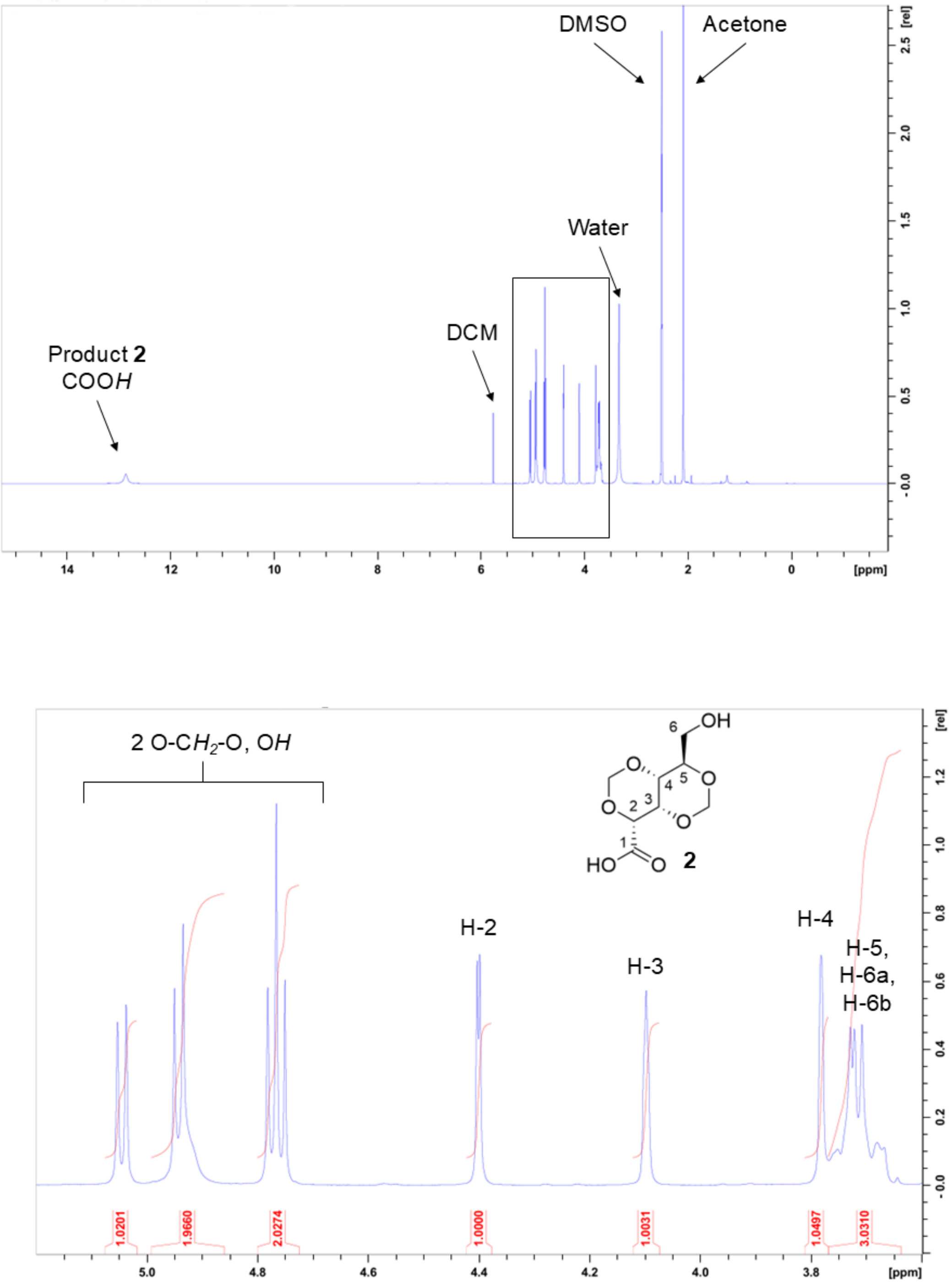

Figure S1. ^{1}H-NMR spectra of 2,4:3,5-Di-*O*-methylene-D-gluconic acid (**2**) in DMSO-d6. Top**:** full range spectrum (solvent peaks are due to residual solvent traces); Bottom**:** zoomed spectrum of product **2**, with full assignments.

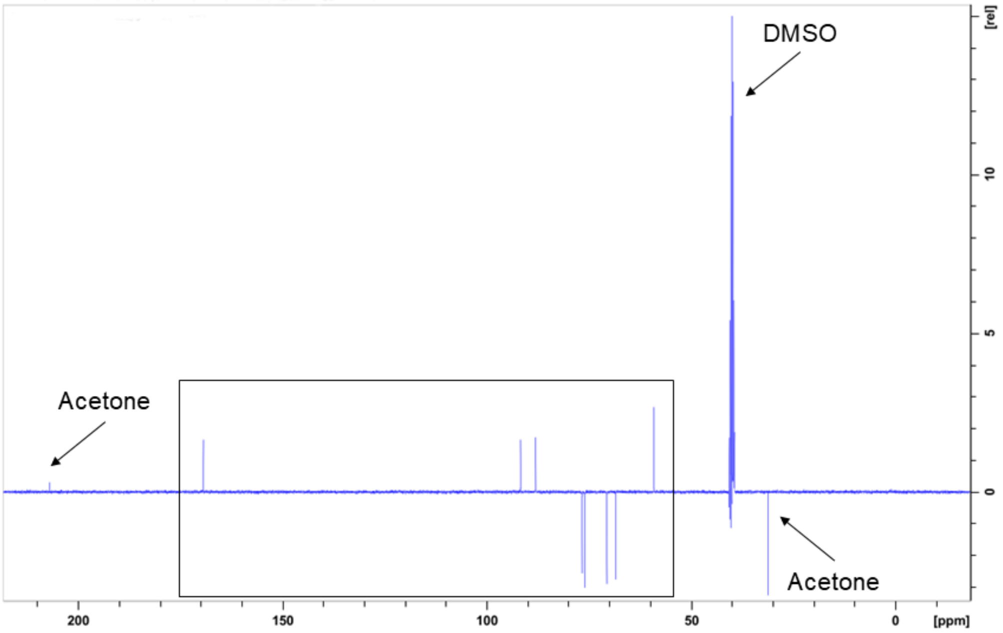

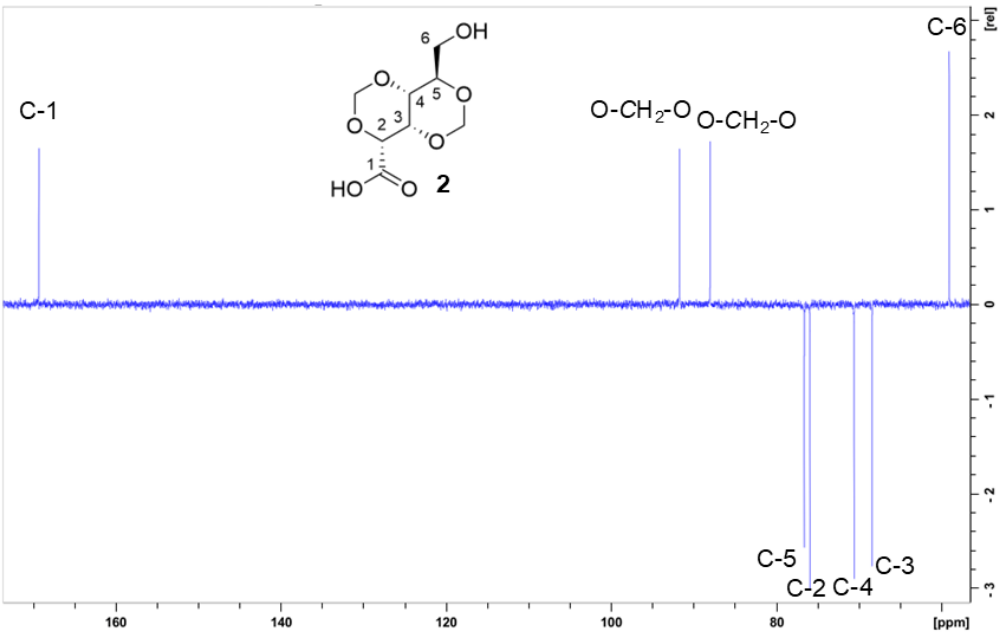

Figure S2. ^{13}C-NMR spectra of 2,4:3,5-Di-*O*-methylene-D-gluconic acid (**2**) in DMSO-d6. **Top:** whole spectrum, solvent peaks are due to residual solvent traces. **Bottom:** zoomed spectrum of product **2**, with full assignments.

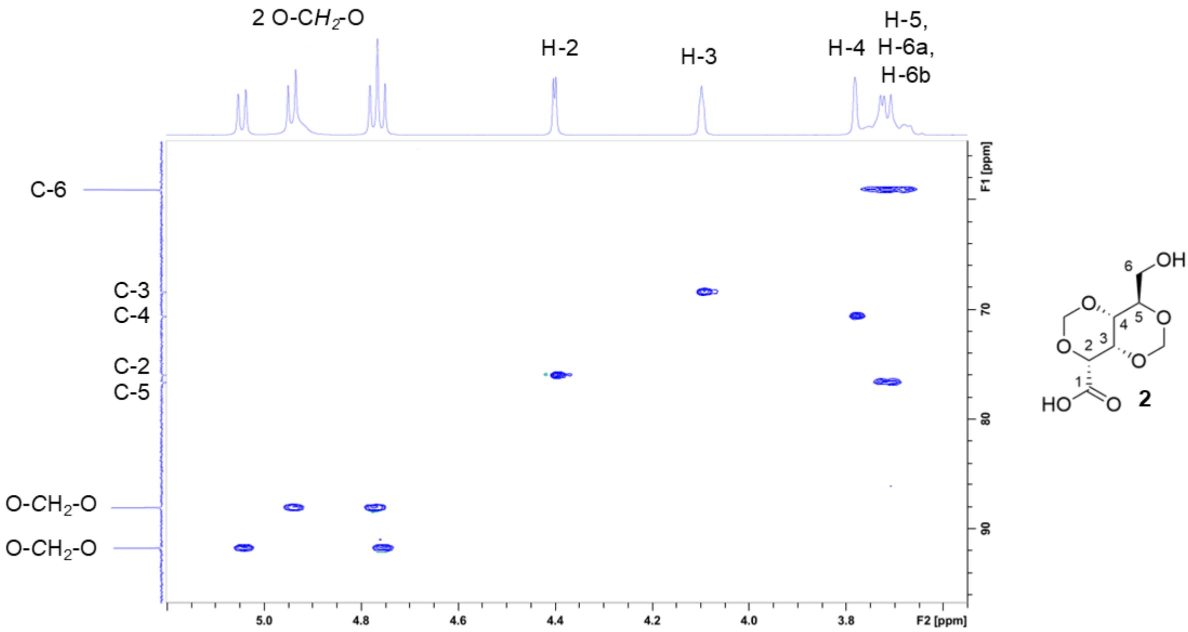

Figure S3. HSQC spectrum of 2,4:3,5-Di-*O*-methylene-D-gluconic acid (**2**) in DMSO-d6.

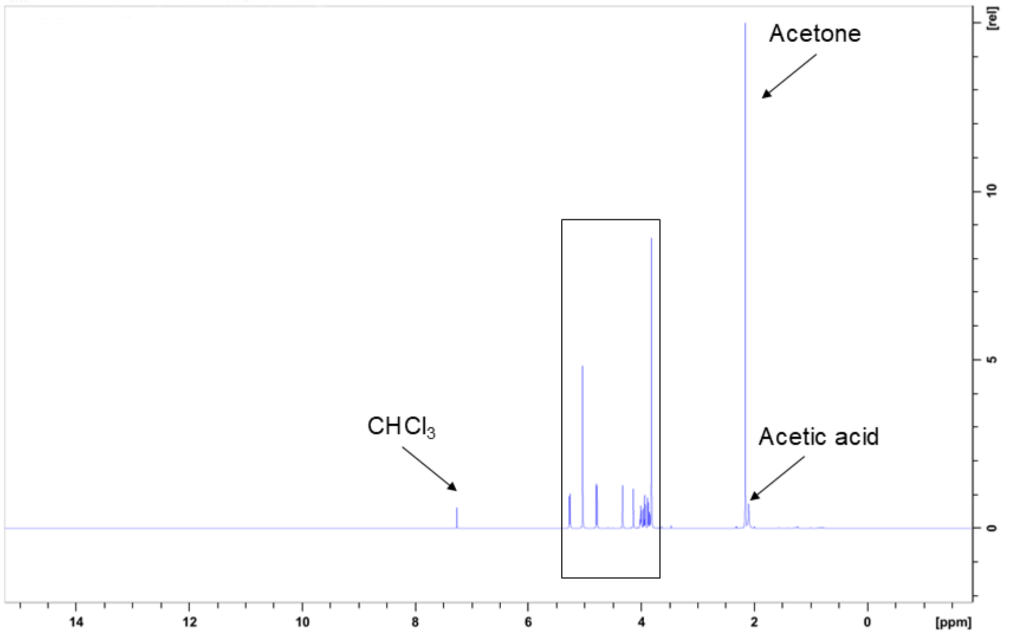

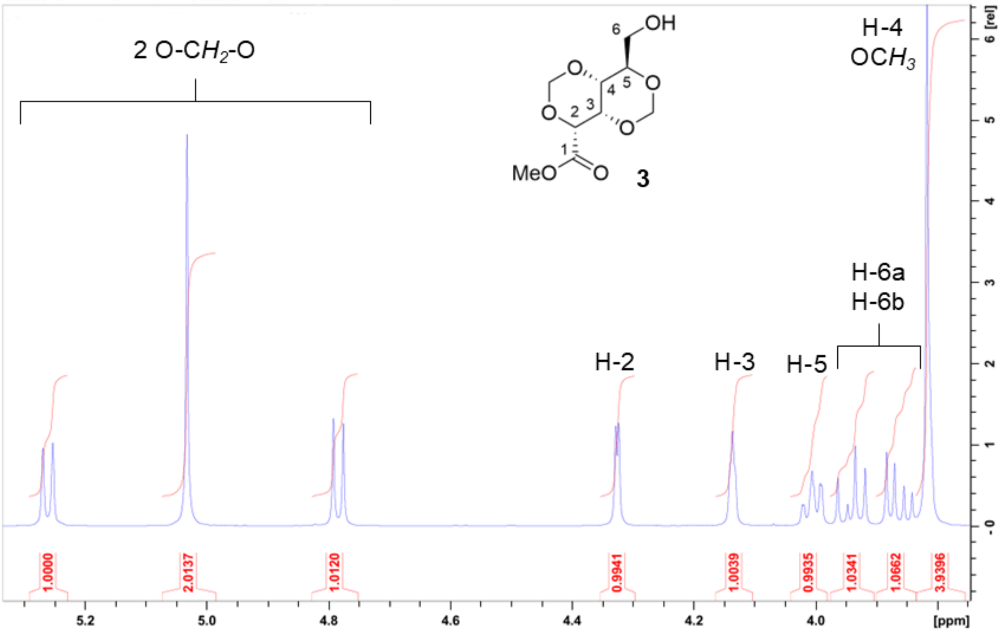

Figure S4. ^{1}H-NMR spectra of methyl 2,4:3,5-di-*O*-methylene-D-gluconate (**3**) in $CDCl_3$. **Top:** whole spectrum, solvent peaks are due to residual solvent traces. **Bottom:** zoomed spectrum of product **3**, with full assignments.

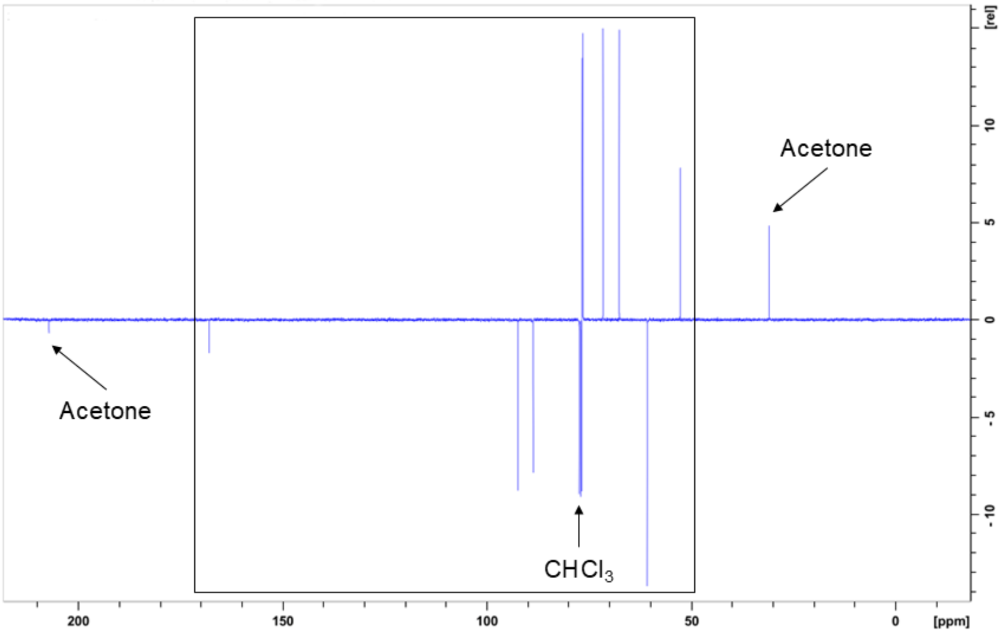

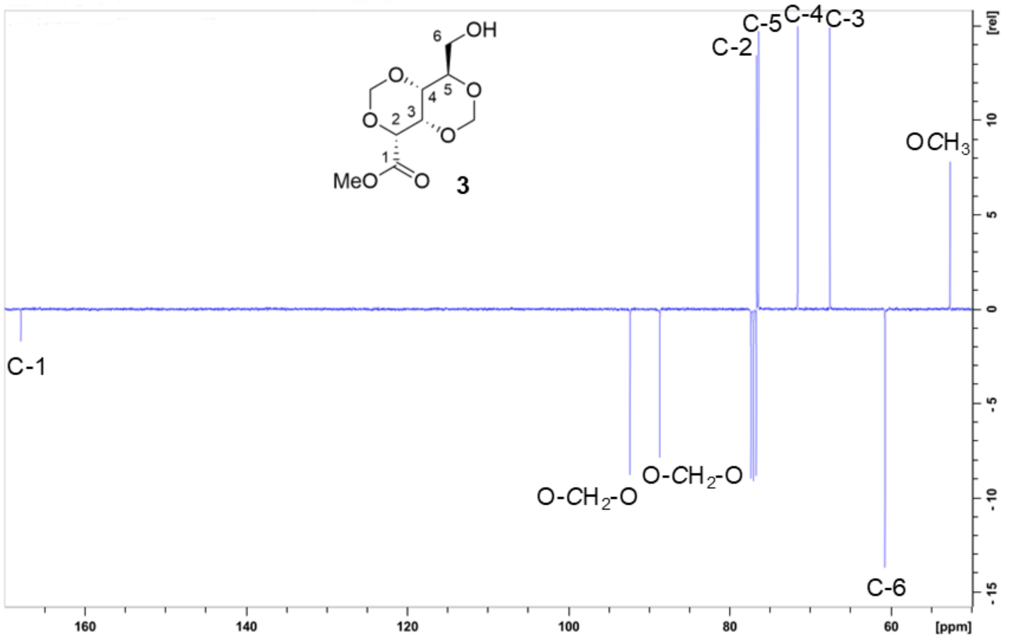

Figure S5. ^{13}C-NMR spectra of methyl 2,4:3,5-Di-*O*-methylene-D-gluconate (**3**) in $CDCl_3$. **Top:** whole spectrum, solvent peaks are due to residual solvent traces. **Bottom:** zoomed spectrum of product **3**, with full assignments.

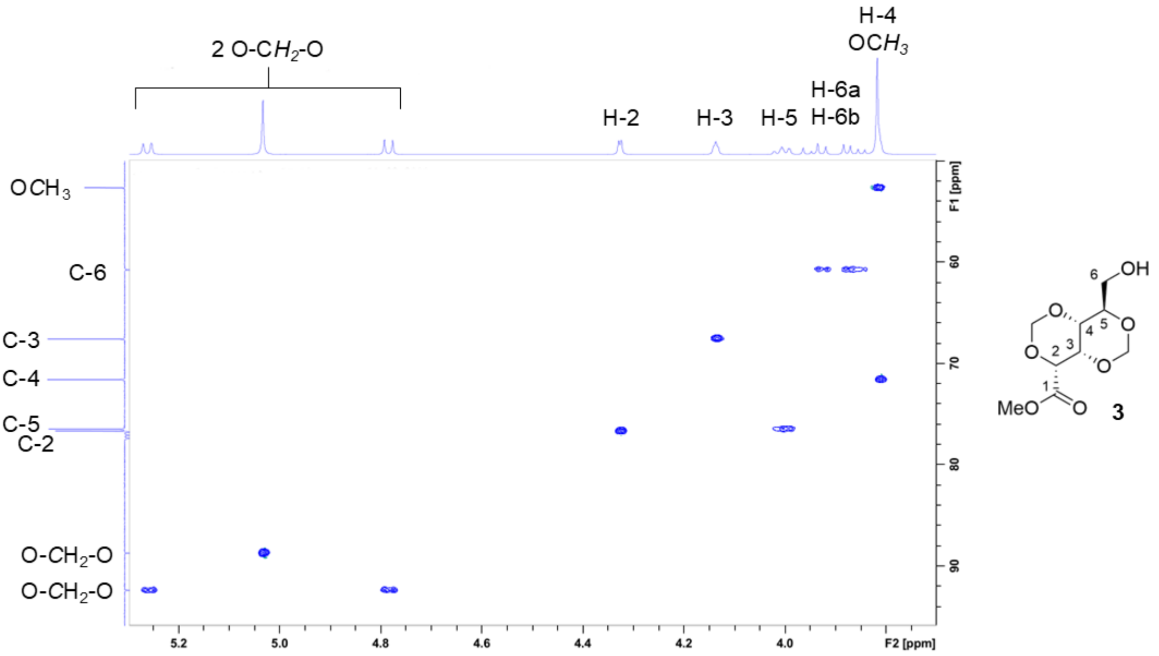

Figure S6. HSQC spectrum of methyl 2,4:3,5-Di-*O*-methylene-D-gluconate (**3**) in $CDCl_3$.

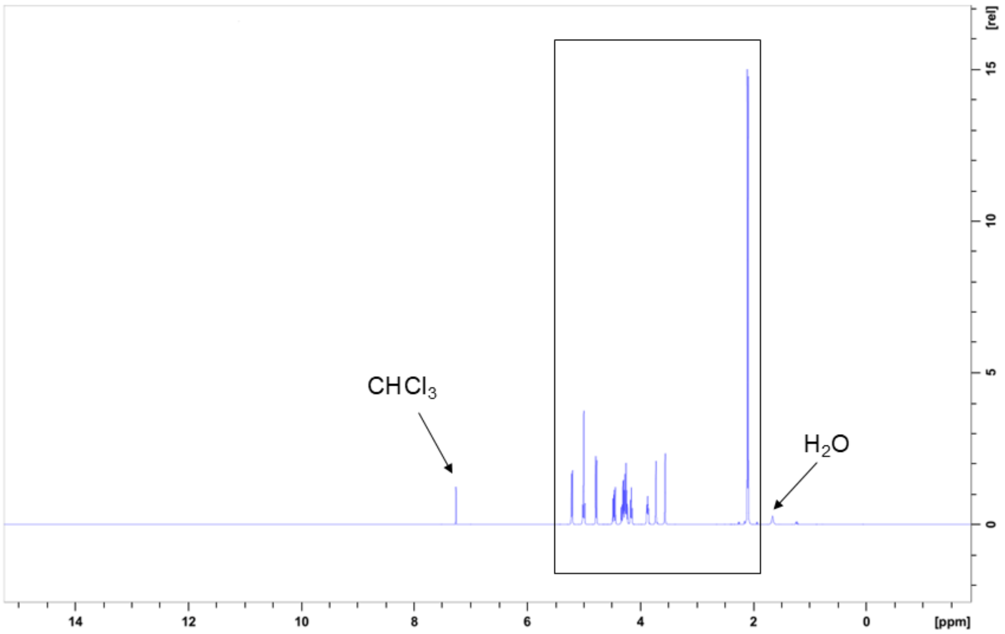

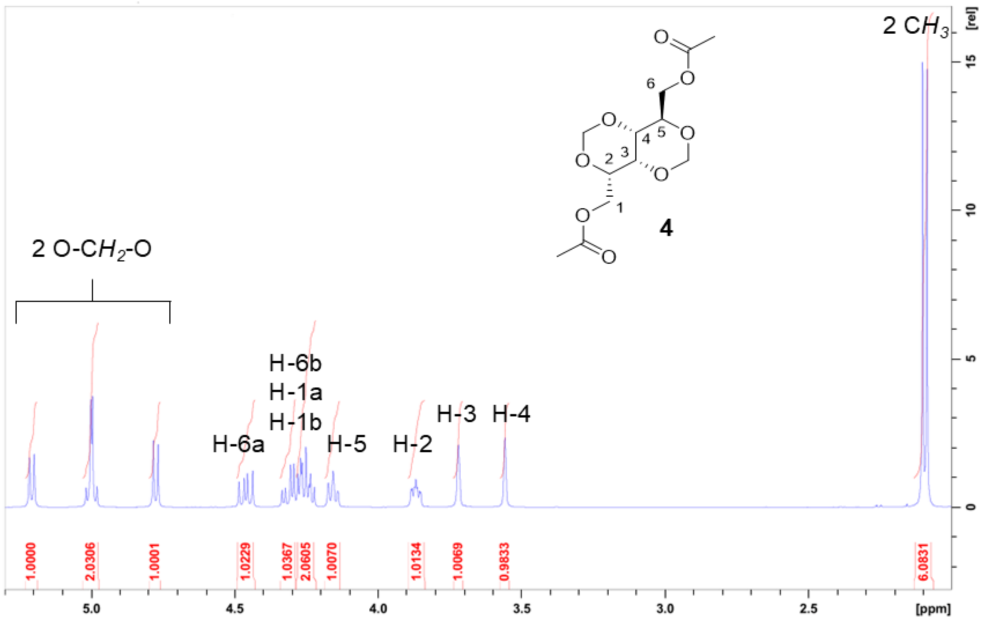

Figure S7. ^{1}H-NMR spectra of 1,6-Diacetyl-2,4:3,5-di-*O*-methylene-D-glucitol (**4**) in $CDCl_3$. **Top:** whole spectrum, solvent peaks are due to residual solvent traces. **Bottom:** zoomed spectrum of product **4**, with full assignments.

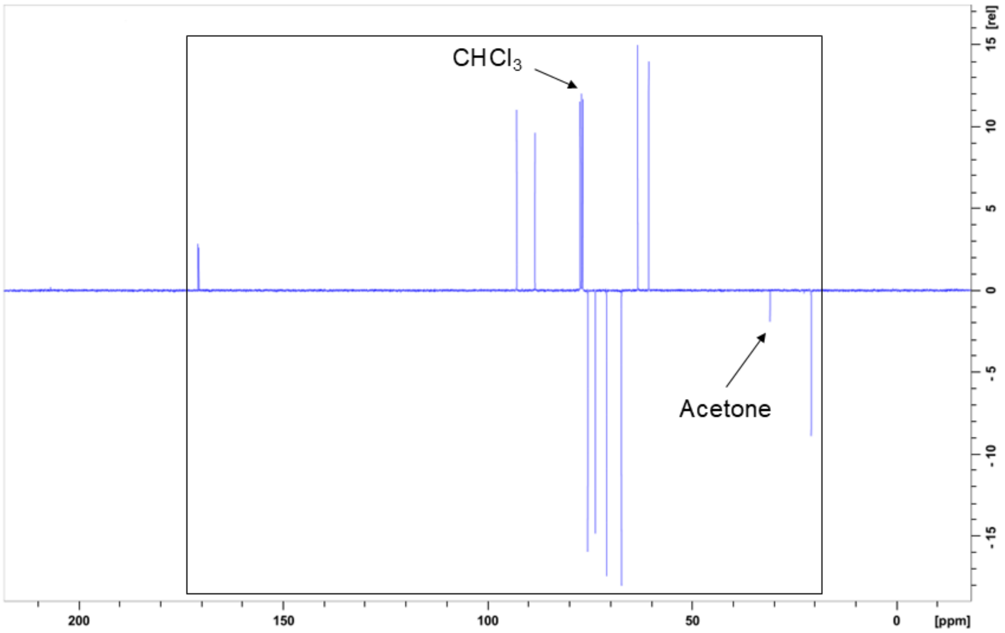

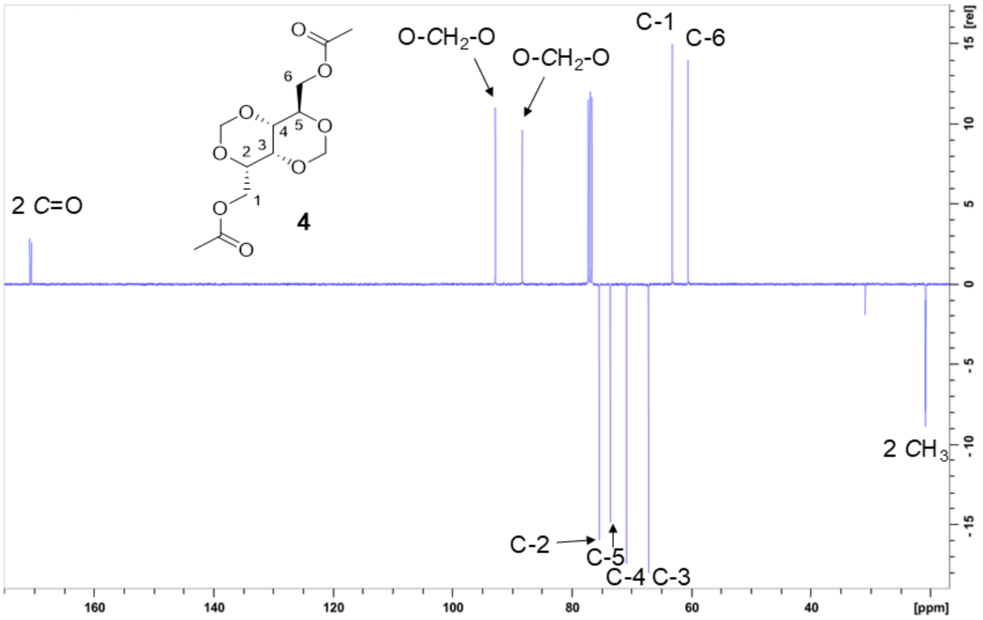

Figure S8. ^{13}C-NMR spectra of 1,6-Diacetyl-2,4:3,5-di-*O*-methylene-D-gluconate (**4**) in $CDCl_3$. **Top:** whole spectrum, solvent peaks are due to residual solvent traces. **Bottom:** enlarged spectrum of product **4**, with compound signal and full assignments.

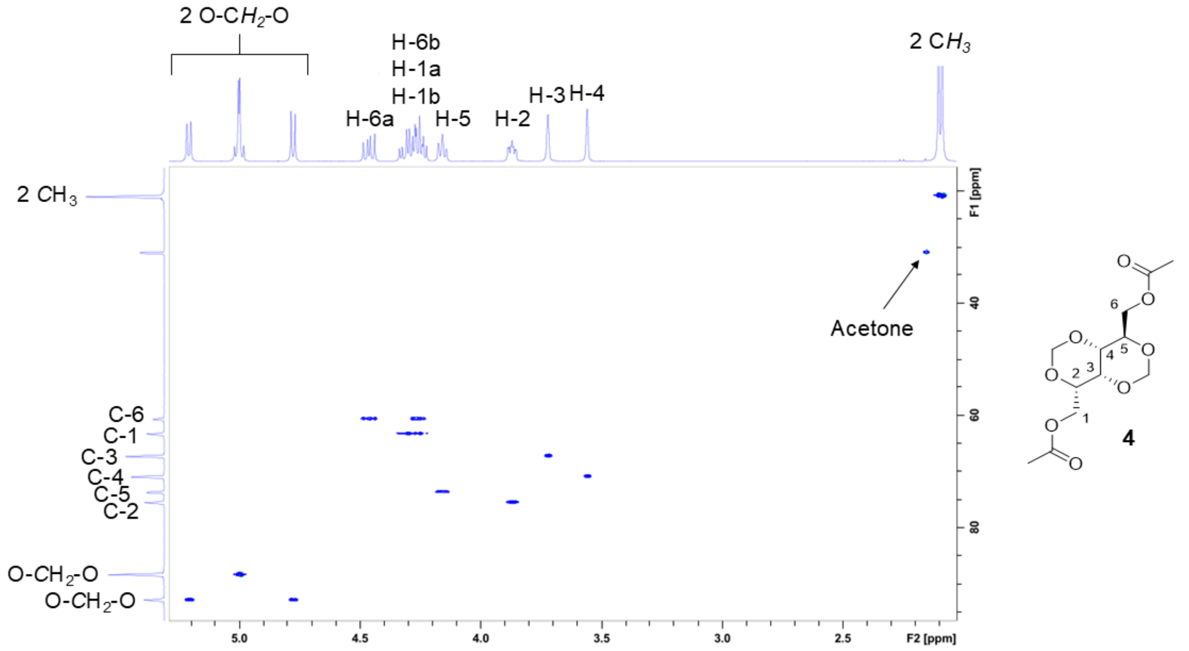

Figure S9. HSQC spectrum of 1,6-Diacetyl-2,4:3,5-di-*O*-methylene-D-gluconate (**4**) in $CDCl_3$, with full assignments. Solvent signals are due to residual solvent traces.

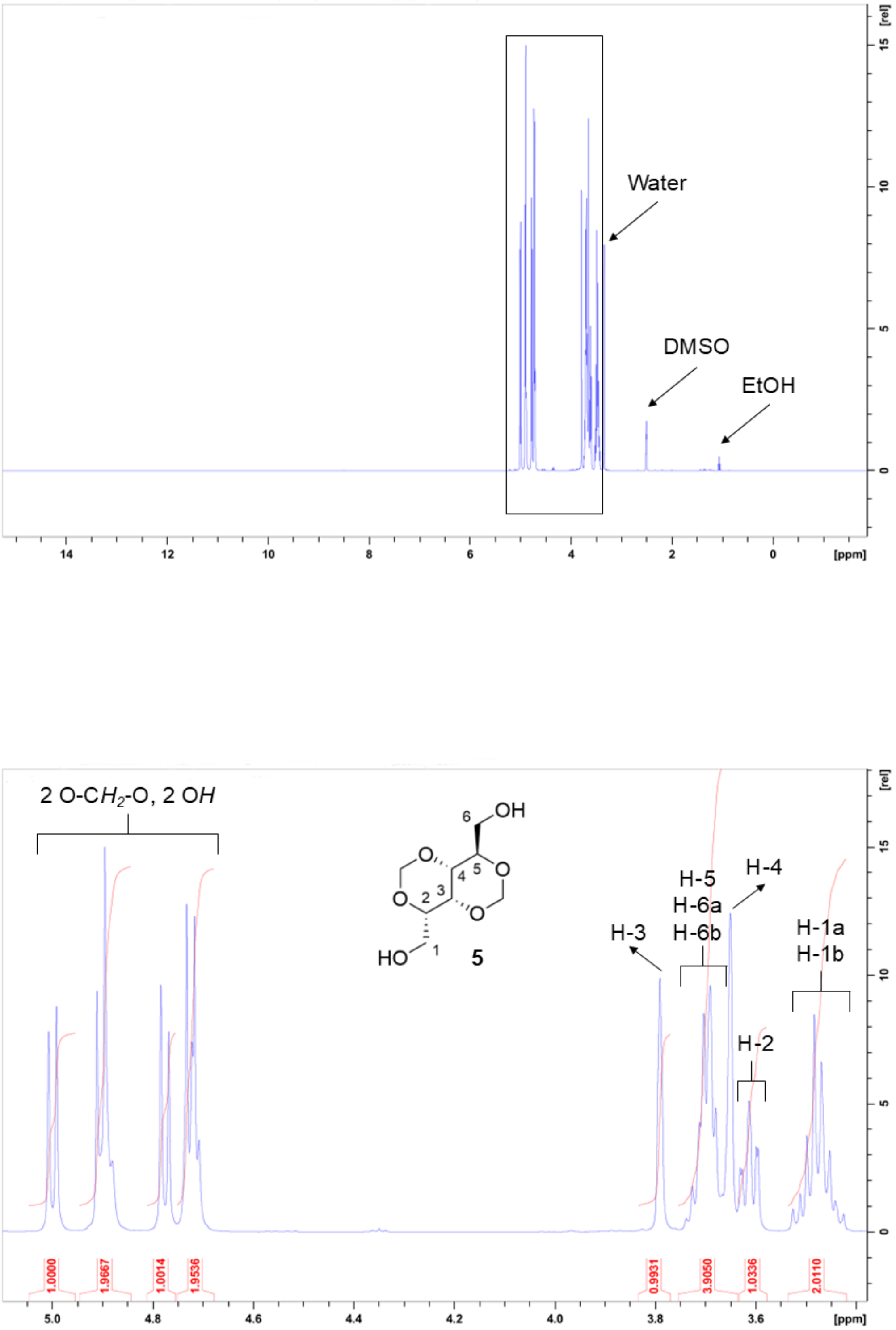

Figure S10. ^{1}H-NMR spectra of 2,4:3,5-Di-*O*-methylene-D-glucitol (**5**) in DMSO-d6. **Top:** whole spectrum. **Bottom:** zoom spectrum of product **5**, with full assignments.

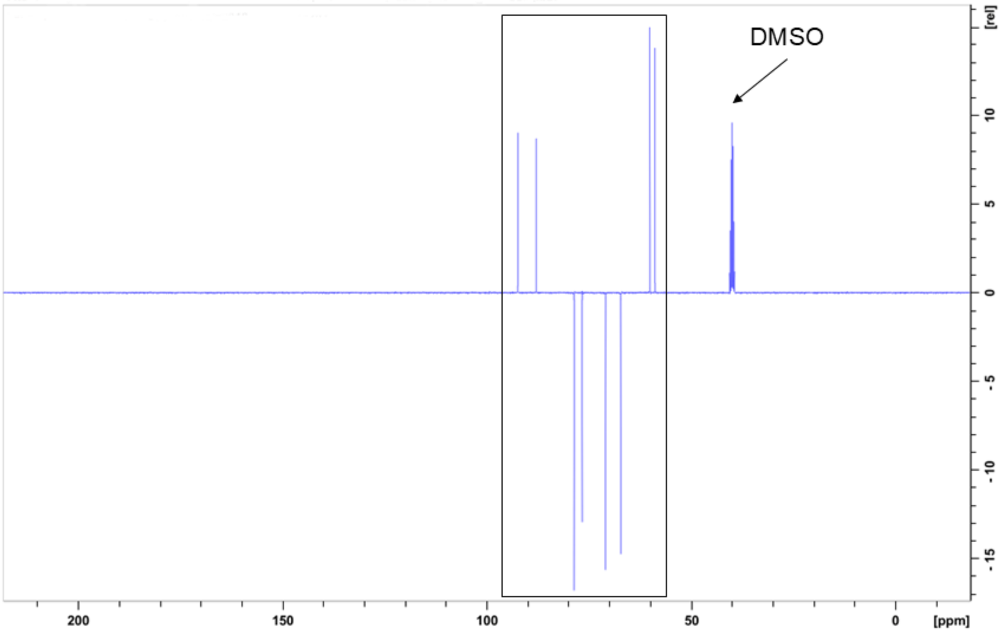

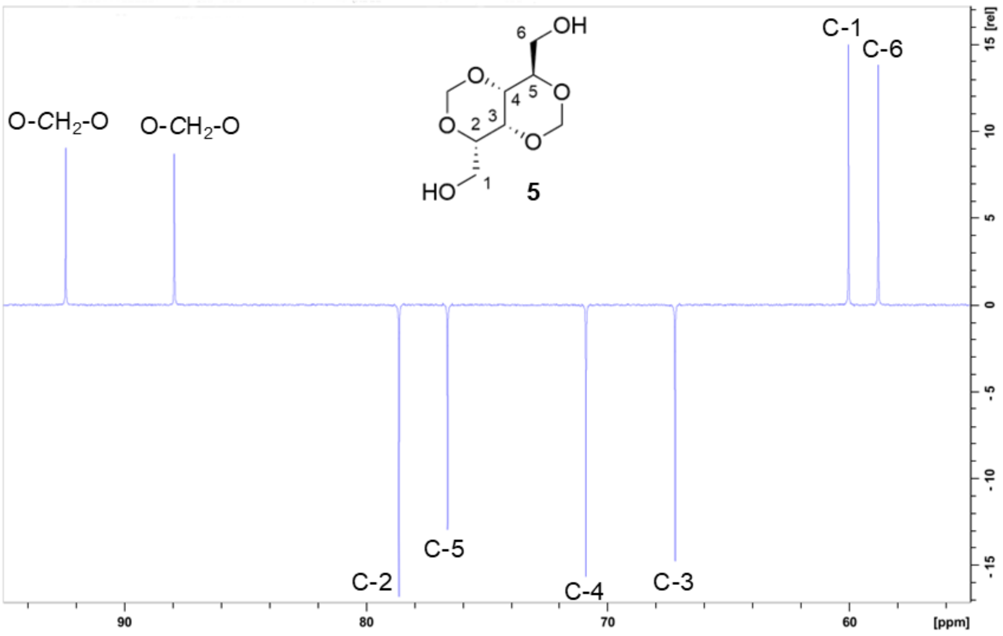

Figure S11. ^{13}C-NMR spectra of 2,4:3,5-Di-*O*-methylene-D-glucitol (**5**) in DMSO-d6. **Top:** whole spectrum. **Bottom:** zoom spectrum of product **5**, with full assignments.

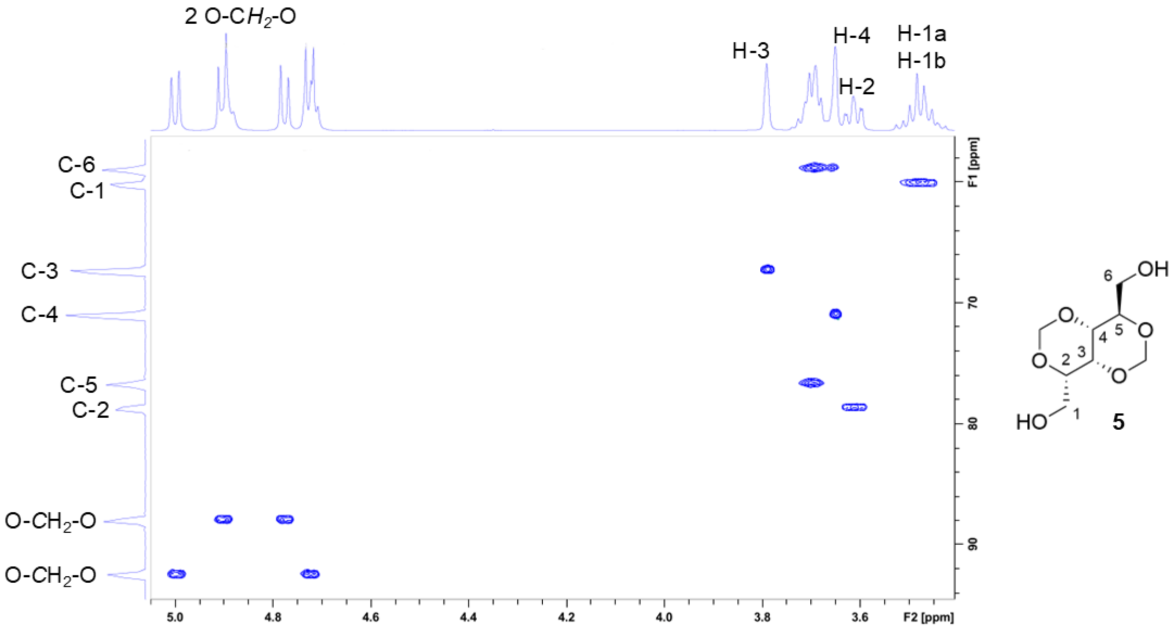

Figure S12. HSQC spectrum of 2,4:3,5-Di-*O*-methylene-D-glucitol (**5**) in DMSO-d6, with full assignments.

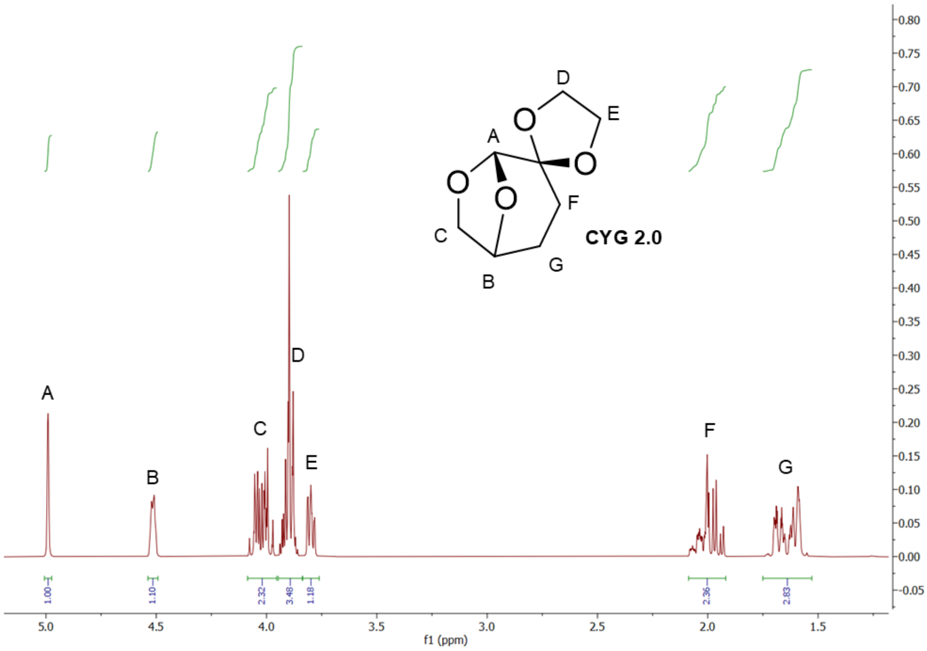

Figure S13. ^{1}H-NMR spectra of Cygnet 2.0 in $CDCl_3$.

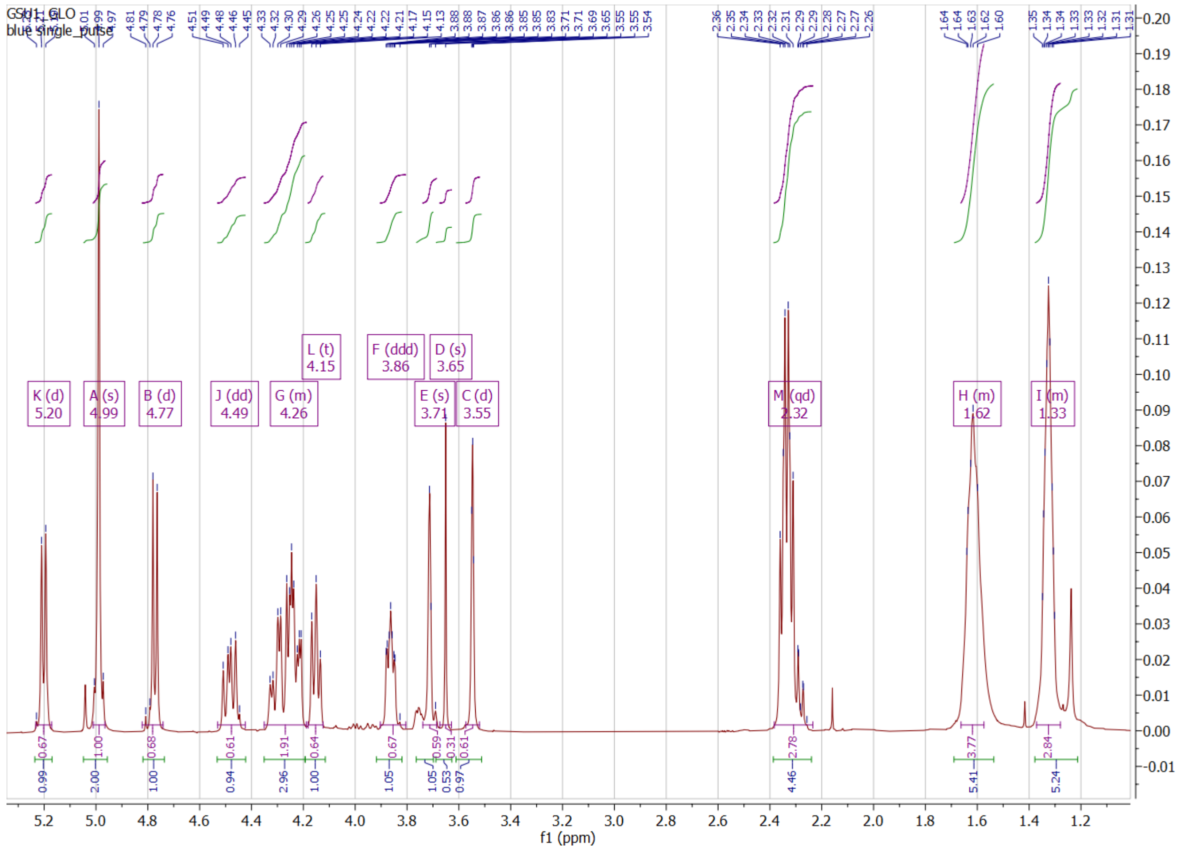

Figure S14. ^{1}H-NMR spectra of Glux-diol and dimethyl succinate (DMS) based polyester in $CDCl_3$.

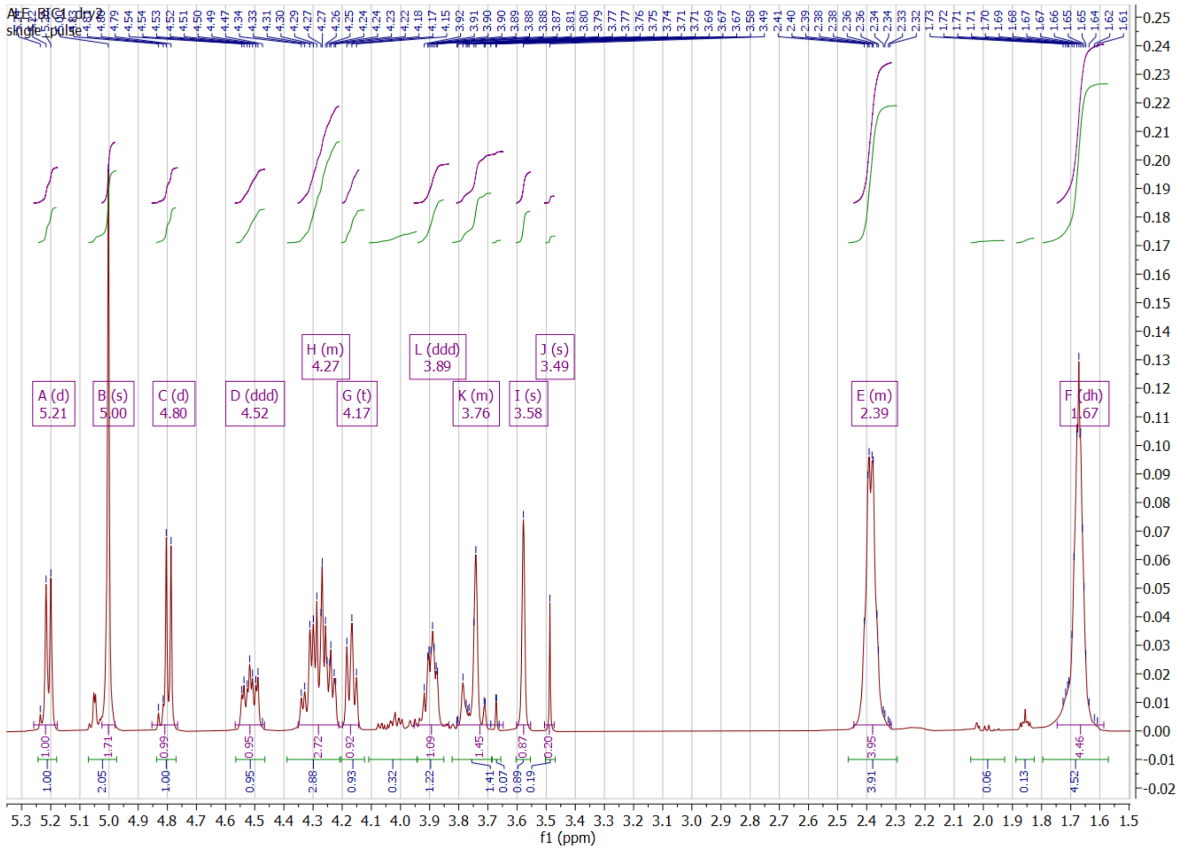

Figure S15. ^{1}H-NMR spectra of Glux-diol and dimethyl adipate (DMA) based polyester in $CDCl_3$.

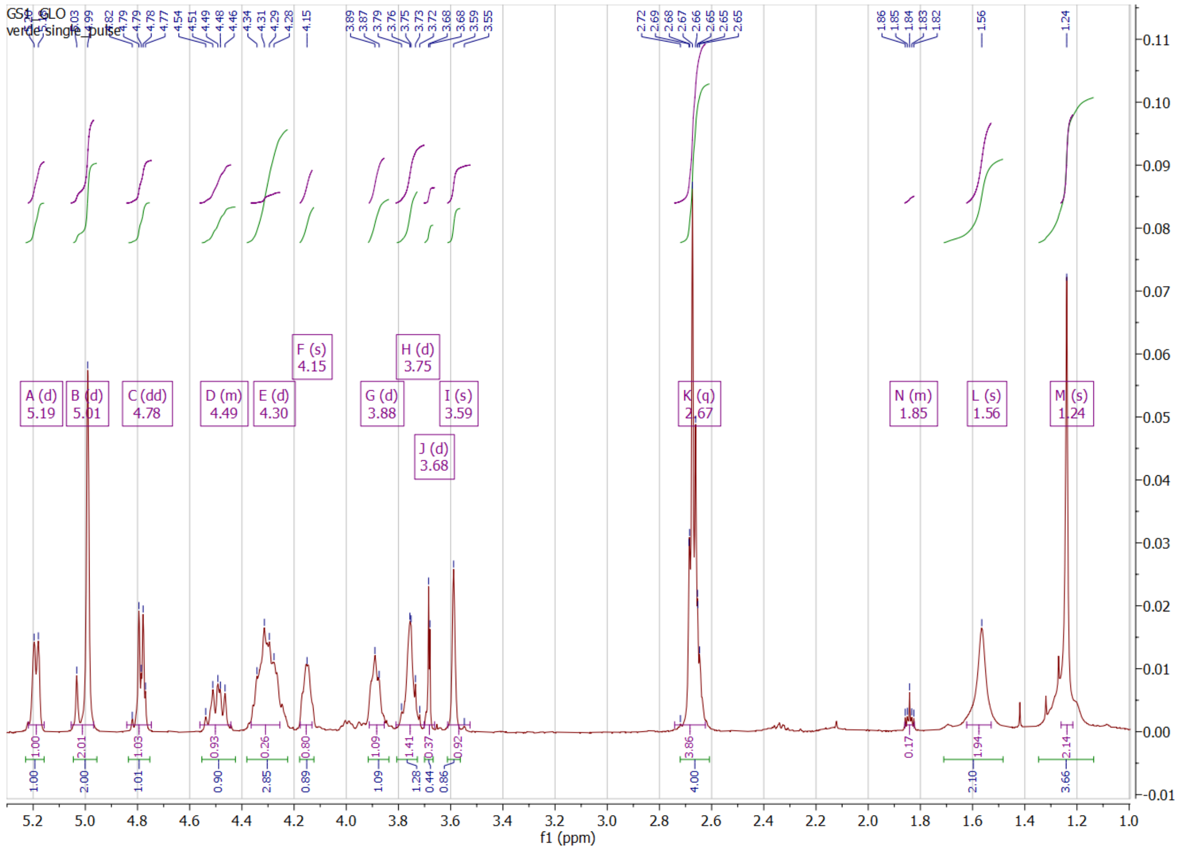

Figure S16. ^{1}H-NMR spectra of Glux-diol and dimethyl suberate (DMSu) based polyester in $CDCl_3$.

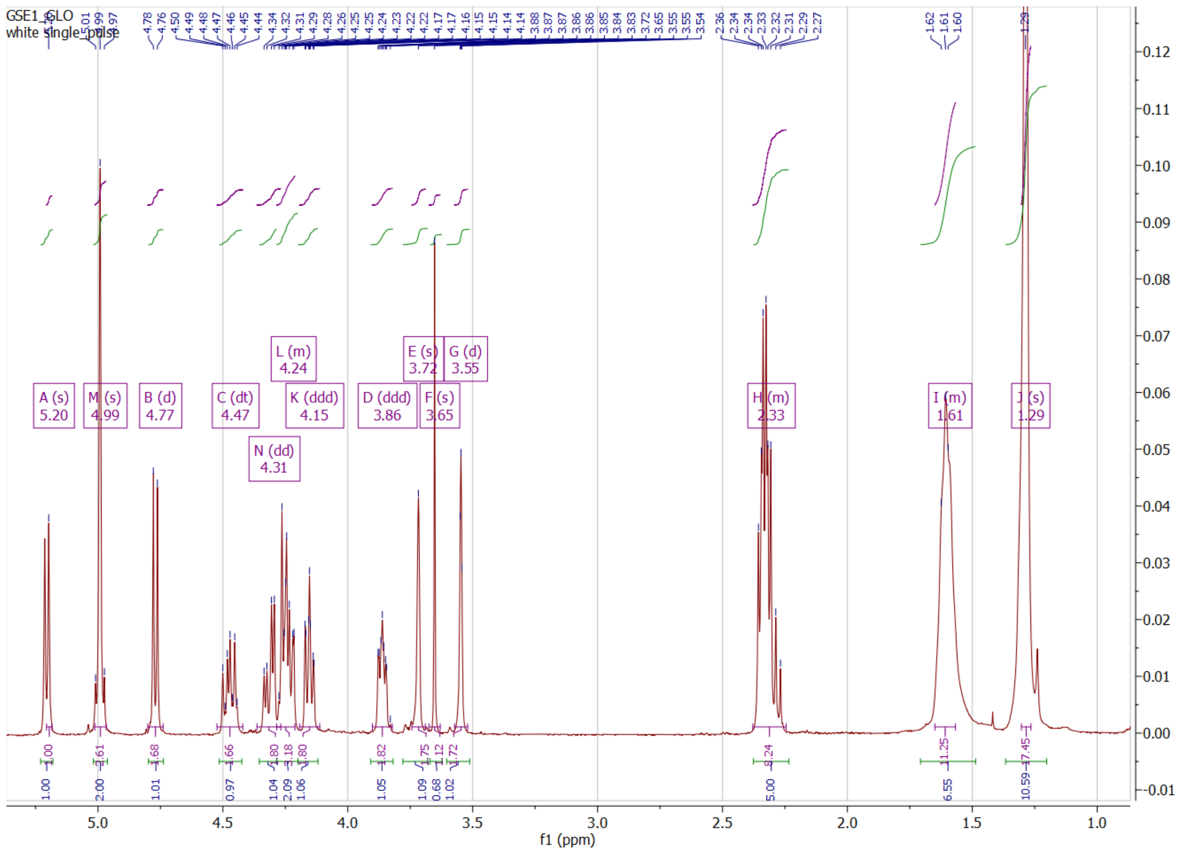

Figure S17. ^{1}H-NMR spectra of Glux-diol and dimethyl sebacate (DMSe) based polyester in $CDCl_3$.

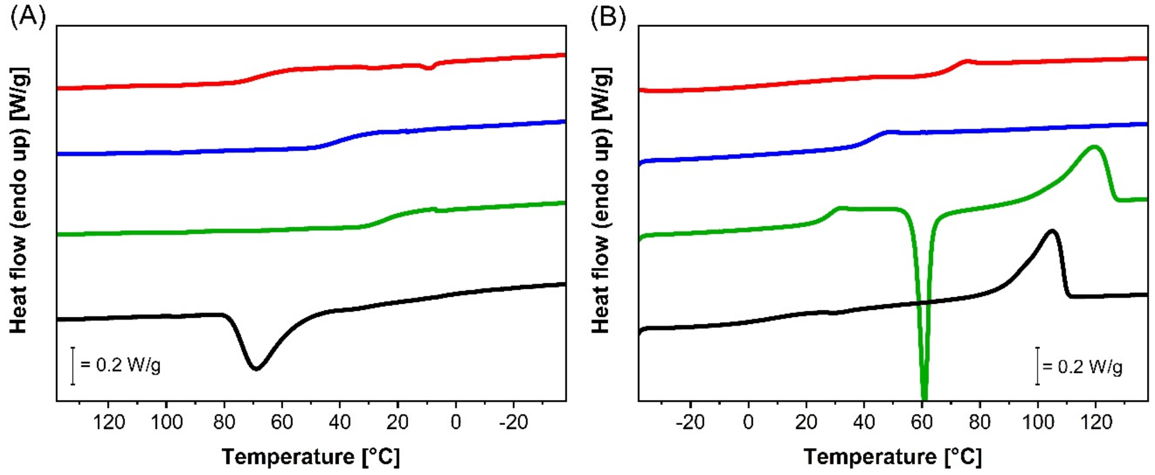

Figure S18. DSC thermograms of glux-diol-based polyesters during cooling (A) and second heating (B) phase prepared with: DMS (red), DMA (blue), DMSu (green), DMSe (black).

[1] Haworth, W. N., & Wiggins, L. F. (1944). 23. The dibenzoates of sorbitol and mannitol and their methylene derivatives. *Journal of the Chemical Society (Resumed)*, 58-61. DOI: 10.1039/JR9440000058

[2] Zief, M., & Scattergood, A. (1947). The Structure of 2,4;3,5-Dimethylene-D-gluconic Acid[1]. *J. Am. Chem. Soc. 1947, 69, 9, 2132*. https://doi.org/10.1021/ja01201a021

[3] Abert M, Mora N, Lacombe JM. Synthesis and surface-active properties of a new class of surfactants derived from D-gluconic acid. Carbohydr Res. 2002;337(11):997-1006. doi:10.1016/s0008-6215(02)00083-6

[4] Marin, R., & Muñoz-Guerra, S. (2009). Carbohydrate-based poly (ester-urethane) s: A comparative study regarding cyclic alditols extenders and polymerization procedures. J. Appl. Polym. Sci., 114(6), 3723-3736. https://doi.org/10.1002/app.30924

[5] Mehltretter, C. L., Mellies, R. L., Rist, C. E., & Hilbert, G. E. (1947). Dimethylene-D-gluconic Acid. *Journal of the American Chemical Society*, *69*(9), 2130-2131.

[6] The Fieser workup for a $LiAlH_4$ reduction containing **n** grams of $LiAlH_4$ involves the successive, dropwise addition to the reaction mixture of **n** mL of water, **n** mL of 15 % (w/v) NaOH (aq), and **3n** mL of water. The result is a granular precipitate of aluminium salts that can be filtered off easily. Fieser, L. F.; Fieser, M. Reagents for Organic Synthesis 1967, 581-595. ISBN: 978-0-471-25875-9

[7] Warne, C. M., Fadlallah, S., Whitwood, A. C., Sherwood, J., Mouterde, L. M. M., Allais, F., Georg M. Guebitz, Con R. McElroy, Pellis, A. (2022). Levoglucosenone-derived synthesis of bio-based solvents and polyesters. Green Chemistry Letters and Reviews, 16(1). https://doi.org/10.1080/17518253.2022.2154573

[8] Hann, R. M., Wolfe, J. K., & Hudson, C. S. (1944). 2, 4: 3, 5-Dimethylene-D-sorbitol and Some of its Derivatives. *J. Am. Chem. Soc. 1944, 66, 11, 1898–1901.* https://doi.org/10.1021/ja01239a031
